# Phosphoproteomics and Bioinformatics Analyses Reveal Key Roles of GSK-3 and AKAP4 in Mouse Sperm Capacitation

**DOI:** 10.3390/ijms21197283

**Published:** 2020-10-02

**Authors:** Nailis Syifa, Jhih-Tian Yang, Chang-Shiann Wu, Miao-Hsia Lin, Wan-Ling Wu, Cheng-Wei Lai, Sheng-Hsuan Ku, Suh-Yuen Liang, Yu-Chun Hung, Chia-Te Chou, Chien-Sheng Wang, Yasushi Ishihama, Jiahn-Haur Liao, Shih-Hsiung Wu, Tzu-Hua Wu

**Affiliations:** 1 Division of Clinical Pharmacy, School of Pharmacy, College of Pharmacy, Taipei Medical University, Taipei 110, Taiwan; d301106008@tmu.edu.tw (N.S.); qjack222@gmail.com (C.-W.L.); lovemeiforever99@gmail.com (S.-H.K.); ooxxro@gmail.com (Y.-C.H.); b303100044@tmu.edu.tw (C.-T.C.); wangjasontw@gmail.com (C.-S.W.); 2 Pharmacy Department, Faculty of Health Science, University of Muhammadiyah Malang, Malang 65145, Indonesia; 3 Ph.D. Program in Microbial Genomics, National Chung Hsing University and Academia Sinica, Taichung 40227, Taiwan; tai-ji-fox@hotmail.com; 4 Department of Information Management, National Formosa University, Yunlin County 632, Taiwan; cswu@nfu.edu.tw; 5 Graduate School of Pharmaceutical Sciences, Kyoto University, Kyoto 606-8501, Japan; miaohsialin1012@ntu.edu.tw (M.-H.L.); yishiham@pharm.kyoto-u.ac.jp (Y.I.); 6 Institute of Biological Chemistry, Academia Sinica, Taipei 115, Taiwan; wuawan0522@gmail.com (W.-L.W.); syliang@gate.sinica.edu.tw (S.-Y.L.); jiahnhaur@gmail.com (J.-H.L.); 7 Ph.D. Program in Drug Discovery and Development Industry, College of Pharmacy, Taipei Medical University, Taipei 110, Taiwan; 8 Master Program in Clinical Pharmacogenomics and Pharmacoproteomics, College of Pharmacy, Taipei Medical University, Taipei 110, Taiwan

**Keywords:** sperm, capacitation, bioinformatics, mouse, GSK-3, AKAP4, IPA

## Abstract

Protein phosphorylation can induce signal transduction to change sperm motility patterns during sperm capacitation. However, changes in the phosphorylation of sperm proteins in mice are still incompletely understood. Here, capacitation-related phosphorylation in mouse sperms were firstly investigated by label-free quantitative (LFQ) phosphoproteomics coupled with bioinformatics analysis using ingenuity pathway analysis (IPA) methods such as canonical pathway, upstream regulator, and network analysis. Among 1632 phosphopeptides identified at serine, threonine, and tyrosine residues, 1050 novel phosphosites, corresponding to 402 proteins, were reported. Gene heatmaps for IPA canonical pathways showed a novel role for GSK-3 in GP6 signaling pathways associated with capacitation for 60 min. At the same time, the reduction of the abundant isoform-specific GSK-3α expression was shown by western blot (WB) while the LFQ pY of this isoform slightly decreased and then increased. The combined results from WB and LFQ methods explain the less inhibitory phosphorylation of GSK-3α during capacitation and also support the predicted increases in its activity. In addition, pAKAP4 increased at the Y156 site but decreased at the Y811 site in a capacitated state, even though IPA network analysis and WB analysis for overall pAKAP revealed upregulated trends. The potential roles of GSK-3 and AKAP4 in fertility are discussed.

## 1. Introduction

Capacitation is an important physiological prerequisite for the sperm cell acrosome reaction and oocyte fertilization [1]. This principle of capacitation was first introduced by Austin [2] and Chang [3]. During capacitation, various proteins in sperm must undergo posttranslational protein phosphorylation mediated by protein kinases, which is important for many cellular processes [4]. Phosphoproteomics workflows have been applied to identify phosphoproteins, localize specific sites of phosphorylation and quantify the extent of modification at particular sites during processes, including sperm capacitation [5]. The first phosphoproteomics study on sperm capacitation, which was performed by Mandal et al. [6], identified 18 peptides from a 95 kDa human sperm protein (FSP95) that is tyrosine phosphorylated during sperm capacitation. A newly published study involving proteomics followed by bioinformatics reveals the involvement of proteins in specific biological, molecular, and cellular pathways in male infertility [7].

Studies on human sperm incorporating phosphoproteomics followed by bioinformatics have identified many novel phosphosites on different proteins, such as CABYR, AKAP3, and AKAP4 [8,9]. One study on boar based on proteomics coupled with ingenuity pathway analysis (IPA) has provided a model of molecular mechanisms, showing that ODF, SPAG6, and AKAP4 affect sperm motility and fertility [10]. AKAP4 is an ERK1/2 substrate, and induction of capacitation leads to activation of the common ERK and PKA/cAMP signaling pathways [11]. Furthermore, cAMP/PKA in turn plays a role in regulating GSK-3 activity by modifying sperm PP1γ2 activity [12], and Wnt signaling can be partially responsible for GSK-3 activity regulation in epididymal sperm [13]. Although some studies coupling phosphoproteomics with bioinformatics have revealed the networks of interacting proteins with upregulated phosphosites, the molecular function during sperm capacitation needs to be investigated in depth.

From a molecular point of view, sperm capacitation has been well studied in vitro and in several species, such as bovines, humans, rats, and hamsters; however, the best-characterized model is the mouse [14], which serves as a de facto surrogate model for the characterization of human sperm capacitation [15]. Limited studies have used phosphoproteomics tools to explore signaling pathways involved in mouse sperm capacitation [16,17]. Previously, 55 unique sites of phosphorylation and 42 different phosphopeptides [17] were identified in mouse capacitated sperm. In 2014, Chung et al. [16] identified 62 distinct phosphotyrosine sites corresponding to 45 proteins with 55 novel phosphotyrosine sites associated with mouse sperm capacitation using a phosphoproteomics approach. Since then, no additional studies have employed phosphoproteomics for the assessment of mouse sperm capacitation, and integrated, advanced bioinformatics tools need to be used to elucidate the signaling pathways that regulate the capacitation process.

In this study, we first performed phosphoproteomics followed by bioinformatics analyses of mouse sperm capacitation. We enriched phosphopeptides with TiO_2_ and subjected them to LC-MS/MS analysis with a label-free quantification (LFQ) approach to compare the phosphoproteomes of noncapacitated and capacitated sperms. We further performed bioinformatics analyses of sperm capacitation using IPA methods for the assessment of information including canonical pathways, diseases and functions, upstream regulators, and networks, and we clarified the kinase related to sperm capacitation by western blot analysis. We aimed to identify phosphorylation profiles and perform bioinformatics analysis to understand signaling pathways and proteins related to sperm capacitation.

## 2. Results

### 2.1. Functional Classification

**Figure 1:**
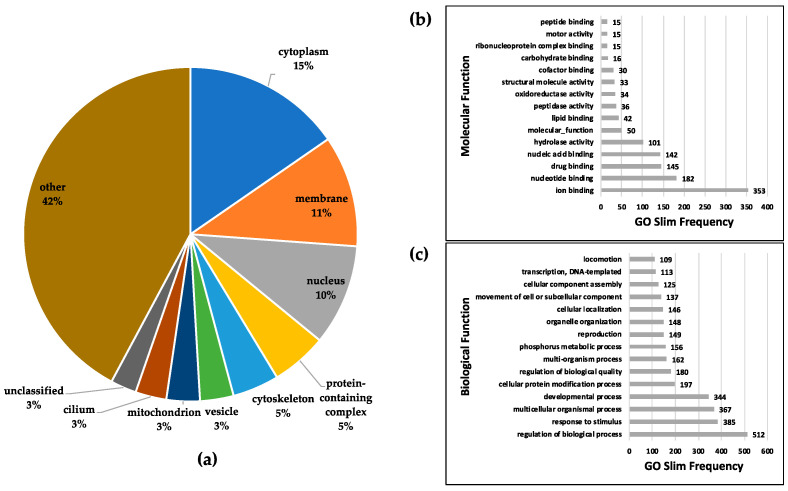
Gene ontology (GO) analysis of the sperm capacitation phosphoproteome. The top 15 selected significant GO slim categories (*p*-value < 0.05) and the ranked frequencies of GO slim categories within the (**a**) cellular component, (**b**) molecular function, and (**c**) biological process gene ontologies are shown. The analyzed phosphoprotein datasets came from all datasets for the three capacitation times (0, 60, and 90 min).

Sperm proteins were extracted using HAMMOC (Hydroxy Acid-modified Metal Oxide Chromatography) phosphopeptide enrichment procedures and analyzed using LC-MS/MS, and the obtained raw data files were then searched with MaxQuant algorithms. The identified phosphorylated proteins were then analyzed by submitting the data to the Protein Information Resource (PIR) website (http://pir.georgetown.edu/) to obtain information on gene ontology (GO) slims relating to sperm capacitation within the cellular component, molecular function, and biological process gene ontologies. In the cellular component category, 15% of the identified proteins were located in the cytoplasm, while 11% and 10% were located in the membrane and nucleus, respectively (Figure 1a). The details of GO analysis of the sperm capacitation phosphoproteome within the cellular component categories are shown in Supplementary Data 1. In the category of molecular function, ion binding (GO:0043167) and nucleotide binding (GO:0000166) were significantly represented (Figure 1b). In the category of biological function, regulation of biological process (GO:0050789), response to stimulus (GO:0050896), multicellular organismal process (GO:0032501) and developmental process (GO:0032502) were the most represented terms (Figure 1c).

### 2.2. Determining Phosphoproteomic Profiling among Datasets

**Figure 2:**
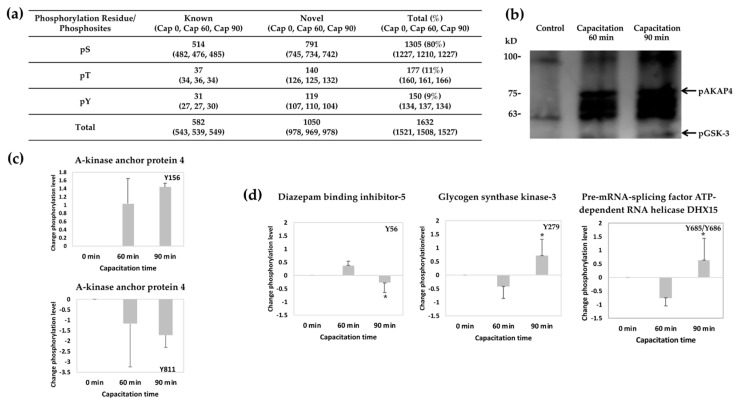
Profiling of protein phosphorylation during mouse sperm capacitation. (**a**) Numbers of phosphopeptides with probabilities > 0.75 for S, T, and Y phosphorylation identified during different capacitation times (0, 60, and 90 min). (**b**) Western blot analysis of Y-phosphorylated mouse sperm proteins after different capacitation times (0, 60, and 90 min) with a pY1000 antibody. (**c**) Label-free quantitation (LFQ) of mouse sperm AKAP4 before or after BSA-induced capacitation. (**d**) LFQ of phosphopeptide level changes from those at capacitation time zero after BSA-induced capacitation. The normalized ion intensities of Y-phosphorylated phosphopeptides corresponding to identified phosphoproteins are plotted versus capacitation time (*X*-axis). * indicates significant differences between the changes at time 60 vs. time 90 (*p* < 0.05).

LFQ phosphoproteomic analysis of noncapacitated and capacitated sperm showed that 3177 phosphopeptides corresponding to 943 proteins were detectable with MaxQuant. Among those, a total of 1632 phosphopeptides including serine (S), threonine (T), and Y residues had phosphosite probabilities > 0.75, and the distribution is shown in Figure 2a. The PhosphoSitePlus^®^ website was searched to identify total known and novel phosphosites that were differentially phosphorylated between noncapacitated and capacitated sperm. These results revealed 1050 novel phosphoserine, phosphotyrosine, and phosphothreonine phosphosites mapped to 402 proteins (Supplementary Data 2).

### 2.3. Detection of Sperm Phosphotyrosine Proteins and LFQ Changes in Y Phosphorylation Following Capacitation

To identify the protein Y phosphorylation changes during mouse sperm capacitation, we performed western blotting using an anti-phosphotyrosine antibody (pY-100) (Figure 2b). The results showed an increase in Y phosphorylation levels after capacitation for 60 and 90 min (Cap 60 and Cap 90, respectively). Moreover, the antibody recognized the presence of proteins with molecular weights of 82 and 50 kDa in capacitated sperm, which were considered phosphorylated AKAP4 and GSK-3 proteins. LFQ was then performed to determine the expression patterns of individual Y-phosphorylated sites during capacitation. Phosphopeptide level changes after bovine serum albumin (BSA)-induced capacitation revealed by LFQ were plotted as the normalized ion intensity of each Y-phosphorylated phosphopeptide versus capacitation time, particularly for the protein identified at three of the capacitation times. The intensities of pAKAP4 at the Y156 site increased and pAKAP4 at the Y811 site decreased during capacitation (Figure 2c), while there were statistically significant differences between the 60 min and 90 min time points for three protein Y phosphorylation intensities, including diazepam-binding inhibitor-like 5, glycogen synthase kinase-3, and pre-mRNA-splicing factor ATP-dependent RNA helicase DHX15 (*p* < 0.05), as shown in Figure 2d. Three other Y phosphorylation changes were observed but were not statistically significant and are summarized in Supplementary Data 3; these changes involved outer dense fiber protein 1, fibrous sheath-interacting protein-2 (FSIP2), and hexokinase-1.

### 2.4. Canonical Pathways and Disease and Function Analysis

To understand the functional relevance of sperm phosphorylation-mediated global phosphoproteomic changes, IPA was used to analyze independent datasets of non-capacitation (Cap 0) in comparison to Cap 60 and Cap 90. This step was followed by comparison analyses of all datasets (Cap 60/0, Cap 90/0, Cap 90/60). The comparison analyses of multiple experimental groups allowed us to identify similarities, differences, and trends.

**Figure 3:**
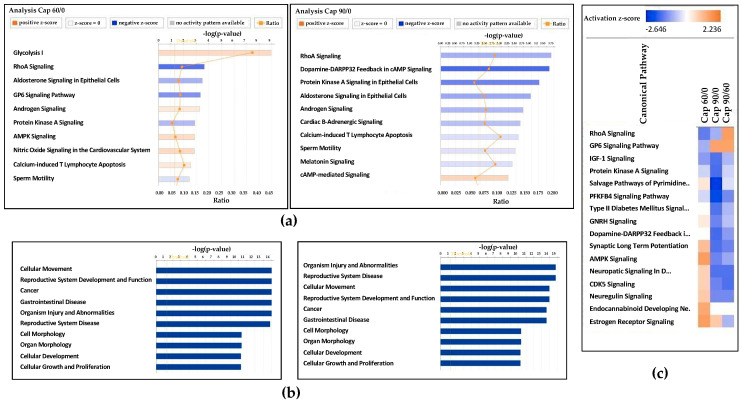
Differential phosphoproteomic analysis using ingenuity pathway analysis (IPA) for the Cap 60/0 and Cap 90/0 datasets. (**a**) Representative bar chart of the IPA-revealed canonical pathways for the Cap 60/0 and Cap 90/0 datasets. The orange lines represent the ratios of changed genes to the total number of genes in specific pathways. The threshold (set to 1.3) is scored as the −log *p*-value from Fisher’s exact test and indicates the minimum significance level. The ratio indicates the number of molecules in the dataset that mapped to the pathway listed divided by the total number of molecules that mapped to the canonical pathway within the IPA database. (**b**) Representative bar charts determined by IPA showing the biological functions associated with the phosphoproteins for the Cap 60/0 and Cap 90/0 datasets. (**c**) Heatmaps generated through IPA canonical pathway analysis for comparison among all datasets (Cap 60/0, Cap 90/0, and Cap 90/60). Upregulated pathways are shaded orange, and downregulated pathways are shaded blue. The intensity indicates the degree to which each gene was upregulated or downregulated as determined by the IPA-determined z-score.

Independent IPA canonical pathway analysis of Cap 60 phosphorylation compared to control (Cap 0) phosphorylation showed altered differential phosphorylation of proteins involved in signaling pathways such as the glycolysis I pathway, the RhoA signaling pathway, the aldosterone signaling pathway in epithelial cells, the GP6 signaling pathway, and the androgen signaling pathway. Similarly, in the Cap 90 dataset, the differential regulation of phosphoproteins was related to canonical pathways including the Rho A signaling pathway, dopamine-DARPP32 feedback in the cAMP signaling pathway, the protein kinase A (PKA) signaling pathway, and the aldosterone signaling pathway in epithelial cells (Figure 3a). The independent disease and biological function analysis revealed the overlapping top pathways in the Cap 60 and Cap 90 datasets, including the cellular movement, reproductive system development and function, reproductive system disease, and organismal injury and abnormality pathways (Figure 3b). Furthermore, gene heatmaps for some major sperm capacitation-associated pathways, including the RhoA, GP6, IGF-1, and PKA signaling pathways and the salvage pathways of pyrimidine, were generated for all phosphorylation datasets (Figure 3c). Of interest, IPA canonical pathway analysis revealed the different expressions of GSK-3 phosphorylation involved in the GP6 signaling pathway (Supplementary Data 4) which has never been reported in a sperm capacitation study. The proteins GSK-3 and PTK2 involved in these pathways were significantly altered between the groups.

### 2.5. Upstream Regulators and Network Analysis

**Figure 4:**
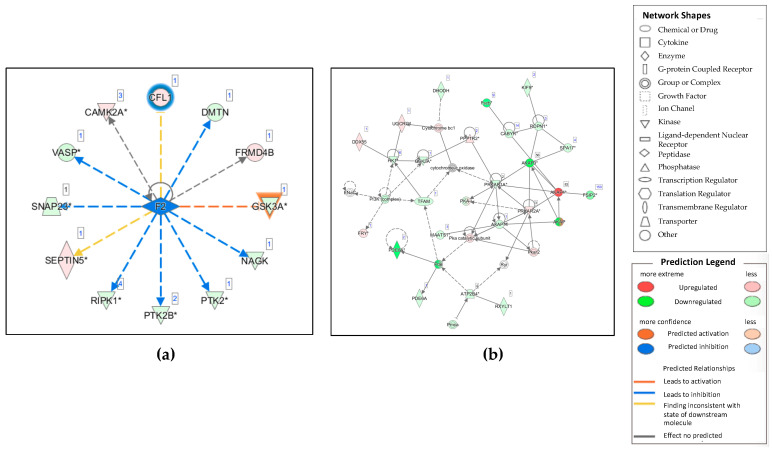
(**a**) Upstream regulators and their corresponding inhibition as predicted by IPA. F2 was predicted to be inhibited (z-score −2.147 and *p*-value 0.011). In this picture, activated upstream regulators are highlighted in orange, while inhibited upstream regulators are in blue. Colors in red and green indicated upregulated and downregulated proteins, respectively, and the color depth is correlated to the fold change. Dashed lines with arrows in orange and blue indicate indirect activation and inhibition, respectively. Yellow dashed lines with arrows indicated inconsistent effects, while gray dashed lines with arrows indicated no prediction. (**b**) IPA-based network of proteins involved in cell signaling, cellular movement, and reproductive system development and function for the Cap 60/0 datasets. In the figure, red represents upregulation and green represents downregulation. The color intensity represents the relative magnitude of the change in protein expression. Direct and indirect interactions are indicated by solid and dashed lines, respectively.

Upstream analysis was performed via IPA to predict the activated or inhibited upstream regulators. The top upstream regulators identified for Cap 60 and Cap 90 were involved in different functions and included the two proteins, integrin alpha-V/beta-3 (ITGB3) and F2. F2 of capacitation 60 was predicted to be significantly inhibited in the Cap 60 group (z-score −2.147 and *p*-value 0.011) (Figure 4a).

Using the network analysis tool within IPA, we predicted interacting molecular networks to further evaluate the related regulatory and/or effector pathways associated with sperm capacitation. Through IPA analysis of the Cap 60/0 and Cap 90/0 comparison datasets, 25 protein networks were predicted. The most significant network functions related to sperm capacitation in the disease and function category were cell signaling, cellular movement, and reproductive system development and function for the Cap 60/0 datasets (Figure 4b). Accordingly, AKAP4, PP1R2, PRKAR2A, and FRY were upregulated, while CABYR, ROPN, GSK-3A, PDE8A, Fscb, KIF9, PRKAR1A, AKAP3, AKAP1, and SPA17 were downregulated.

### 2.6. Validation of Identified Proteins by Western Blotting

**Figure 5:**
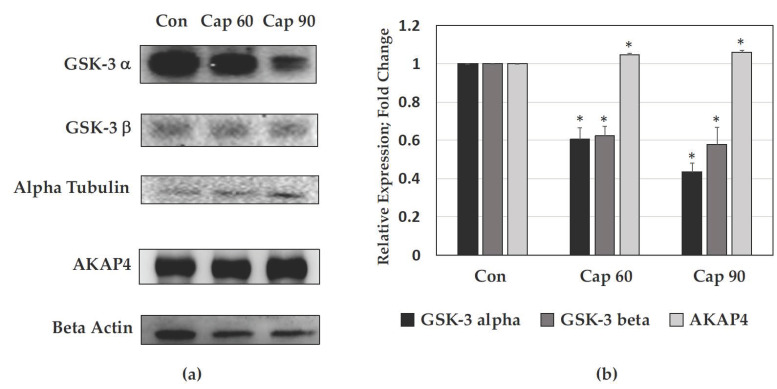
Validation of MS results by western blot analysis for selected proteins. (**a**) Western blot analysis of GSK-3α, GSK-3β, alpha tubulin, AKAP4, and beta actin in noncapacitated (Con) and capacitated (Cap 60 and Cap 90) mouse sperm. (**b**) Relative expression of GSK-3α, GSK-3β, and AKAP4, (fold changes). * indicates significant differences compared to control (Con) group of each selected protein (*p* < 0.05).

We then performed western blot analysis to validate the expression of two proteins in noncapacitated and capacitated sperm, GSK-3 and AKAP4, in order to understand their roles (Figure 5a,b). The results showed that GSK-3α levels in sperm in the Cap 60 group were 38% lower than those in noncapacitated sperm, while those in sperm in the Cap 90 group were 57% lower than those in noncapacitated sperm. In addition, the GSK-3β values in Cap 60 and Cap 90 sperm were 38% and 42% lower than those in noncapacitated sperm, respectively. On the other hand, the expression of AKAP4 in capacitated sperm was higher than that in noncapacitated sperm.

## 3. Discussion

In this study, we reported 1050 novel phosphopeptides corresponding to 402 proteins that have not been identified before. During mouse sperm capacitation, pAKAP4 increased at the Y156 site and decreased at the Y811 site, while pGSK-3 at the Y279 site significantly decreased in Cap 60 and significantly increased in Cap 90, determined by LFQ. In addition, upregulated AKAP4 protein, which directly interacts with downregulated PRKAR1A kinase, and downregulated GSK-3 kinase, which directly interacts with upregulated PPP1R2 kinase and indirectly interacts with reduced PI3K (complex) in Cap 60/0 datasets, were identified by network analysis and it was therefore newly found that the GSK-3 pathway interacts with the GP6 signaling canonical pathway during capacitation. The protein expression of GSK-3 was decreased in both isoform α and β, but not in AKAP4. How to explain the predicted increased activity observed by IPA with reduced GSK-3 α and β protein expression remains unclear.

According to the GO slim statistics in the sperm phosphoproteome, 15% of the identified proteins are located in the cytoplasm, 11% are located in the membrane, and 10% are located in the nucleus (Figure 1a). Due to the unique morphology of sperm, 3% of the identified proteins, including fibrous sheath CABYR-binding protein, outer dense fiber protein 2, and AKAP4, are located in the cilium. The identified phosphorylated proteins are involved in various cellular processes, such as metabolic processes including nucleotide, carbohydrate, phosphorus, and organic acid metabolism, which may imply that metabolic processes are not as active in sperm as in normal cells. Upon analyzing related cellular processes, we found that proteins associated with the movement of cells or subcellular components and locomotion are related to sperm motility, such as phosphoglycerate kinase 2 [18], sperm mitochondrial-associated cysteine-rich protein [19], sperm-associated antigen 17 [20], AKAP4 [21], and dynein [22]; these proteins are all related to sperm motility.

Quantitative analysis of the proteomics data with MaxQuant revealed 3177 phosphopeptides. Of those, 1632 phosphopeptides had probabilities above 0.75; they were phosphorylated at S, T, and Y residues and mapped to 565 proteins. The 1632 phosphopeptides consisted of mainly phosphorylated S residues (80%), followed by phosphorylated T (11%) and Y (9%) residues (Figure 2a). A previous study identified a total of 3303 phosphorylated sites [8] and 3527 phosphorylated sites [9] in sperm in humans but not in mice. The phosphopeptides identified in this study were then compared with data on PhosphoSitePlus, which provided evidence of 1050 novel phosphosites that have not been previously characterized in mouse sperm (Supplementary Data 2). Among these novel phosphopeptides, some of the proteins were found to be related to the movement of cells, such as AKAP4, outer dense fiber protein 2, fibrous sheath CABYR-binding protein, ropporin-1, and septin-4. Even though the abovementioned proteins have been previously reported to be related to sperm motility [21,23,24,25], we here provide newly identified phosphosites of sperm proteins to further support the roles of these proteins in capacitation.

It is well documented that protein phosphorylation, especially at Y residues, is one of the most important events that occurs during capacitation [1]. In the present work, we analyzed phosphotyrosine changes during sperm capacitation using western blotting. As expected, capacitated sperm showed significantly greater Y phosphorylation than noncapacitated sperm (Figure 2b), as supported by a previous study [9,26,27,28]. Moreover, changes in phosphorylation levels during sperm capacitation have been identified in several proteins. The identified phosphopeptides corresponding to the AKAP4 protein are shown in Figure 2c. Among these phosphopeptides, analysis of changes in phosphorylation levels at individual phosphosites revealed a tendency toward upregulation of phosphorylation at Y156 (1.04 in Cap 60; 1.44 in Cap 90) after capacitation, while phosphorylation at Y811 (−1.18 in Cap 60; −1.71 in Cap 90) was downregulated during capacitation. A previous phosphoproteomics study in mice conducted by Platt et al. [17] revealed AKAP4 phosphorylation at three different sites (S226, S65, and S812) with odds ratios (ORs) of 4.61, 3.27, and 2.29, respectively, while Chung et al. [16] reported phosphorylation at three different sites of AKAP4 (Y292, Y138, and Y438) with normalized ratios of 4.1, 2.3, and 1.4, respectively. All these observations further support our hypothesis that Y156 or Y811 phosphorylation events are novel and may play different roles during mouse sperm capacitation. In addition to the phosphotyrosine sites of AKAP4, glycogen synthase kinase-3 (Y279) and diazepam-binding inhibitor-like 5 (Y56) (Figure 2d), which are involved in sperm energy metabolism, have been reported to show significantly different phosphorylation levels at similar phosphosites in a previous study [16]. Although there were no significant differences in the phosphorylation levels at the phosphotyrosine site (Y623) of FSIP2, this site was identified as a new phosphosite (Supplementary Data 3); a previous study reported different sites (Y630, Y1635, Y6101, and Y6351) [16]. In the sperm principal piece, the fibrous sheath supports signaling proteins that regulate motility, capacitation, and hyperactivation [29]. The major components of the sheath are AKAP3 and AKAP4, which probably form the integral cytoskeleton structure [30]. Hexokinase-1, which is also involved in energy metabolism, showed changes in Y phosphorylation at the Y83 site in the current study (Supplementary Data 3), consistent with a previous study [16,17]. Taken together, these findings suggest that immunoaffinity precipitation with a specific phosphorylation motif antibody can complement the current methodology to obtain a comprehensive view of the phosphoproteome.

Next, to achieve a comprehensive understanding of the system-wide phosphoproteome data, IPA was used to analyze all datasets, which elucidated many differentially phosphorylated proteins associated with sperm capacitation. As stated in the results, the significant biological functions altered in the Cap 60 and Cap 90 datasets were regulation of cellular movement and reproductive system development and function (Figure 3b). Canonical pathway analysis showed regulation of the glycolysis I signaling pathway (−log *p*-value 8.95) in the Cap 60 datasets and RhoA signaling (−log *p*-value 3.66) in the Cap 90 datasets. Comparison analysis of all datasets showed alterations in many signaling pathways, most importantly the RhoA signaling pathway, the GP6 signaling pathway, the IGF-1 signaling pathway, and the PKA signaling pathway (Figure 3c). The involvement of RhoA signaling in bovines [31] and guinea pigs [32]; IGF-1 signaling in humans [8,33] and bovines [34]; and PKA signaling in mice [35] and bovines [36] during sperm capacitation has been previously reported, while that of the GP6 signaling pathway has never before been reported.

GSK-3 in the GP6 signaling pathway showed alterations during sperm capacitation in the present study (Supplementary Data 4). According to our IPA results regarding the GP6 signaling pathway, GSK-3 was activated in the Cap 90/0 and Cap 90/60 comparison, while in the Cap 60/0 comparison, GSK-3 was inhibited; thus, GSK-3 was inhibited or phosphorylated in the Cap 60 dataset. A previous phosphoproteome study showed abundant GSK-3α phosphorylation in high-motility human sperm [37]. Li’s study demonstrated that H2AX phosphorylation can be abolished by PI3K inhibition and therefore rescue DNA in spermatozoa from oxidative damage [38]. GSK-3 may also contribute to identifying markers for DNA damage since its substrates are regulated via the PI3K-AKT-GSK-3 (Figure 4b) signaling network [39]. In fact, DNA integrity analysis is a better diagnostic and prognostic marker of sperm reproductive potential, for example, changes in nuclear basic proteins in human sperm exposure to heavy metals were recently explored [40] to link to DNA damage. The current study aimed to explore translational protein markers specific to the process of capacitation to mimic sperm cells passing to the uterus. In the future, the association of the targeted protein phosphorylations with the modulated DNA damage via H1 histones will be determined. Furthermore, Vadnais proposed a sperm motility cascade through the PDPK1-AKT1-GSK-3 pathway [41]. To our knowledge, the current study is the first study to show the involvement of the GP6 signaling pathway in sperm capacitation mediated by PDPK-AKT-GSK-3 (Supplementary Data 4). Notably, GP6 is required for collagen-induced platelet activation [42], while platelet-activating factor (PAF) can affect the capacitation, acrosome reaction, and fertilization potential of sperm [43,44,45,46]. The current IPA results provide a framework for future experiments regarding the involvement of the GP6 signaling pathway in sperm capacitation.

IPA revealed no evidence that F2, officially named coagulation factor II (prothrombin), is related to sperm capacitation. The downregulated genes GSK-3A, NAGK, PTK2, PTK2B, RIPK1, SNAP23, DMTN, and VASP led to the inhibition of F2 protein (Figure 4a). Among these proteins, GSK-3A, protein kinase C epsilon (PRKCE), PTK2, and PTK2B were previously reported to be involved in sperm regulation. PRKCE is involved in the regulation of flagellar motility in human sperm [47]. Inhibition of protein tyrosine kinase 2 (PTK2 or FAK) during capacitation affects the protein Y phosphorylation associated with capacitation, which causes the acrosome reaction to become increasingly Ca2+ dependent and inhibits the polymerization of actin [48]. Protein tyrosine kinase 2B (PTK2B or PyK2) is an intermediary component of Ca2+ signaling between PKA-mediated and Y phosphorylation that is required for achieving functional human sperm capacitation [49]. Various upstream regulators that are chemical components, such as bisindolylmaleimide I upstream regulators, were predicted to be significantly activated (z-score 2.401). Bisindolylmaleimide I is a protein kinase C inhibitor involved in sperm function that decreases calcium influx and the acrosome reaction in noncapacitated and capacitated sperm is induced by progesterone [50]. Catsper is a sperm-specific low voltage-dependent calcium channel that was identified in mouse sperm in 2001 and only two types, Catsper1 and Catsper2, are highly specialized in mammalian sperm, and are associated with progesterone-induced progressive motility due to Ca^2+^ entry into sperm through the Catsper channel. The crucial function of the SLO3 channel is to balance the membrane hyperpolarization correlates with capacitation through potassium spermospores [51]. A previous study investigated male contraception by targeting the function of calcium channel Catsper1 in sperm [52]. Since bisindolylmaleimide I decreases calcium influx in capacitated spermatozoa through Catsper channel inhibition that can reduce sperm progressive motility, the chemical and its downregulated protein, such as GSK-3 observed in the IPA results, can be proposed as a male contraceptive target in the future.

Further analysis using IPA demonstrated a cell signaling, cellular movement, and reproductive system development and function network consisting of 24 focus molecules in the proteomic datasets (Figure 4b). This network centers on cytochrome bc1 [53], cytochrome-c oxidase [54], PI3K (complex) [55], ENaC (complex) [56], PKA [55], AKAP [35], PKA-I [57], Pkar2 [58], the PKa catalytic subunit [59], PDE (complex) [60], Pmca [61], and Ryr [62], which together mediate signals relevant to cell signaling, cellular movement, and reproductive system development and function. The levels of many proteins were found to be decreased in association with capacitation, such as AKAP3, GSK3A, AKT, ROPN1, CABYR, KIF9, Fscb, FSIP2, DHODH, AKAP1, SPA17, TFAM, MAATS1, PDE6A, ATP2B4, and RXYLT1. Of interest, analysis of the cell signaling, cellular movement, and reproductive system development and function network showed that the abundance of AKAP4 was significantly increased in the Cap 60 dataset.

Validation of the global proteomic findings further strengthened the bioinformatics results. Based on the canonical pathway, upstream regulators, and network revealed by IPA, two proteins (GSK-3 and AKAP4) were selected for validation by western blotting in capacitated and noncapacitated mouse sperm. Western blot analysis (Figure 5) revealed significant reductions in the protein expression of GSK-3α and GSK-3β in capacitated sperm, and IPA upstream regulator analysis (Figure 4a) predicted increased GSK-3α protein activity but decreased phosphorylation in the overall dataset. However, increased Y phosphorylation of GSK-3 is known to increase activity, while S phosphorylation of GSK-3 is known to decrease activity. Hence, in the future, the specific sites of Y and S phosphorylation of GSK-3 during sperm capacitation need to be determined. Hopefully, the greater increase in Y but fewer changes in S phosphorylation of GSK-3α and less inhibitory phosphorylation can be observed and explain the increased GSK-3α protein activity predicted by IPA in the Cap 60 dataset. A previous study in bovines showed increased phosphorylation during capacitation but without details of the two isoforms, or identifying isoform α and β, as well as in the capacitation state [63,64,65] and in human sperm [8,63,66]. Moreover, a porcine study used the similar capacitation medium condition to the current study or GSK-3 inhibitor to induce GSK-3α inactivation by S phosphorylation increase in the isoform GSK-3α, and also observed increases of the motile sperm parameters but did not identify the Y phosphorylation [67], as well as in goat sperm [68]. In humans, isoform-specific GSK-3α serine phosphorylation without assaying activity was increased [66]. Our study is the first to show pGSK-3 at the Y279 site significantly decreased in Cap 60 and significantly increased in Cap 90 and the activity of GSK-3α was predicted to be decreased during mouse sperm capacitation. On the other hand, in Figure 2b and Figure 5, western blot analyses revealed that the protein expression intensity of AKAP4 was significantly increased, which may have resulted from the observed increase in Y phosphorylation during capacitation, which is supported by Figure 4b. AKAP4 is required for glyceraldehyde 3-phosphate dehydrogenase-S to bind to the fibrous sheath and glycolysis, a major source of energy for sperm functions essential for fertility, is disrupted in sperm lacking AKAP4. AKAP4 interacts with fibrous sheath proteins such as CABYR and ropporin, regulating calcium signaling, which is important for sperm capacitation induction [69]. Moreover, the subcellular distributions of PKA catalytic subunits and regulatory subunits, such as PI3K, were disrupted and there were significant changes in PP1gamma2 activity and phosphorylation [70] in immotile sperm from infertile mice lacking AKAP4 [71]. In addition to the 43 novel phosphosites of AKAP4 that have been identified, there were 29 AKAP3 novel phosphosites reported here, and the interactions between those phosphorylation signals will be our future interest.

To our knowledge, this is the first study coupling proteomics with bioinformatics analysis using IPA to investigate the phosphorylation molecular pathways associated with various mouse sperm capacitation times. The current study reveals the changes in GSK-3α/β isoforms and AKAP4 expression that occur during mouse sperm capacitation, contributing to identifying markers related to sperm motility and fertility.

## 4. Materials and Methods

### 4.1. Mouse Sperm Isolation

As previously described in a protocol from our lab [72], ICR mice purchased from Biolasco (I Lan, Taiwan) were bred in the Animal Center at Taipei Medical University according to the protocol (approval numbers LAC-2015-0265, LAC-2018-0345) and animal handling was according to the guidelines. Sperm were taken from the cauda epididymides of 12- to 16-week-old male mice and placed in buffer solution containing 120 mM NaCl, 1.99 mM KCl, 1.06 mM MgSO_4_·7H_2_O, 0.3 mM NaH_2_PO_4_, 5.6 mM d-glucose, 18.4 mM sucrose, 10.9 mM HEPES, 1 M sodium pyruvate, and 1 M NaHCO3. This medium was prepared in the absence of BSA and does not support capacitation. Sperm were incubated at 37 °C under 5% CO_2_ and filtered. After incubation, the sperm were centrifuged at 1750 rpm at room temperature. To induce capacitation, sperm were treated with calcium and BSA and were incubated at 37 °C under 5% CO_2_ for 60 or 90 min.

### 4.2. Protein Digestion

Cell lysates or 0.25 µg/µL β-casein were reacted with 4.5 mM 1,4-dithiothreitol (DTT) at 37 °C for 2 h and then reacted with 11.25 mM iodoacetamide (IAA) at room temperature with protection from light. Next, 25 mM ammonium bicarbonate and trypsin (1:20) were added for trypsin digestion and allowed to react at 37 °C for more than 16 h.

### 4.3. Spiking of the Internal Standard and Phosphopeptide Enrichment

For LFQ, 0.5 µg of digested β-casein peptides were spiked into each sample before desalting with a C18 membrane followed by HAMMOC enrichment [26]. As described in the literature [73,74], phosphopeptides were enriched using HAMMOC with 0.5 mg of TiO_2_ beads (GL Sciences, Tokyo, Japan) packed into 10 µL C8-StageTips. These home-made HAMMOC tips were washed with solution A (0.1% trifluoroacetic acid (TFA) and 80% acetonitrile (ACN)), after which solution B (solution A containing lactic acid (300 mg/mL)) was added as a selectivity enhancer to equilibrate the tips. Each tip contained 100 μg of dry digested sample peptides that had been redissolved in solution A and diluted with an equal volume of solution B before loading. Solutions A and B were used to wash the tips and to remove nonspecifically bound peptides. Sequential elution was performed with 0.5% and 5% piperidine to obtain pure phosphopeptides. The eluted phosphopeptides were acidified in 20% phosphoric acid to pH 2.5, desalted with a 3M Emphore SDB-XC stagetip, concentrated as described above, and subjected to LC-MS analysis [75].

### 4.4. LC-MS/MS Analysis

LC-MS/MS analysis was performed on an LC-ESI-Mass system (Orbitrap Fusion mass spectrometer (Thermo Fisher Scientific, San Jose, CA, USA)). Peptide samples in 0.1% formic acid (FA) were injected onto a self-packed precolumn (150 µm inner diameter (ID) × 30 mm, 5 µm, 200 Å) and a 75-μm × 20 cm fused silica capillary column packed with 2.5-μm C18 beads ReproSil-Pur Basic^®^ (Maisch), in a 250 μL/min gradient of 5% ACN/0.1% FA to 40% ACN/0.1% FA over the course of 40 min, with a total run time of 60 min and a flow rate of 300 nL/min. The Orbitrap Fusion instrument was operated in data-dependent mode to automatically switch between full-scan MS and MS/MS acquisition. Full MS survey scans from *m*/*z* 200 to 1400 were carried out at a resolution of 120,000 using EASY-IC as the lock mass for internal calibration. The MS/MS analysis was run in top-speed mode with 3 s cycles, while the dynamic exclusion duration was set to 60 s with a 25 ppm tolerance around the selected precursor and its isotopes. Monoisotopic precursor ion selection was enabled, and 1+ charge states were rejected from MS/MS. Automatic gain control was employed and set to 2 × 10^5^ for MS. The maximum allowed ionization time was 200 ms. These experiments were carried out with higher collision energy dissociation fragmentation modes for S, T, and Y.

### 4.5. MS Data and Bioinformatics Analysis

The MS-derived data were analyzed using MaxQuant 1.6.1.0 to identify the sites of protein phosphorylation [76]. According to the default parameters of this version of the software, phosphosites with >75% localization probability were considered [77,78]. The amino acid phosphorylation sites from PhosphoSitePlus^®^ (www.phosphosite.org) were used to identify novel phosphosites. The PhosphoSitePlus^®^ database collects comprehensive posttranslational modification information extracted from published data [79]. Quantitative changes in the levels of phosphorylation during sperm capacitation were calculated for several proteins by normalization to the intensity of the internal control β-casein. The identified phosphorylated proteins were then submitted to the PIR website (https://proteininformationresource.org/) [80] for GO slim classification with respect to biological processes, molecular functions, and cellular components. The quantified phosphosites were analyzed using IPA (Qiagen). The IPA program facilitates the evaluation of canonical pathways, diseases and functions, upstream regulators, and signaling networks related to sperm capacitation. The significance of the association between a dataset and a canonical pathway was measured in two ways according to the description in a previous study [78]. Comparison analysis was carried out between the two analyzed datasets (Cap 60/0 and Cap 90/0) and among all datasets (Cap 60/0, Cap 90/0, and Cap 90/60) to identify the differences in the canonical pathways in capacitated sperm.

### 4.6. Verification of the AKAP4 and GSK-3 Proteins by Western Blotting

Briefly, cells were lysed in phosphate-buffered saline containing protease inhibitors and phosphatase inhibitors. The protein concentration was determined using BCA methods. Equal amounts of protein from capacitated and noncapacitated sperm were separated by SDS-PAGE and transferred to PVDF membranes. The membranes were then blocked by incubation with 5% nonfat dry milk diluted in TBST. After blocking, the PVDF membranes were washed with TBST and stained with a primary antibody diluted in 2.5% nonfat dry milk in TBST. The following primary antibodies were used: anti-PY-1000 (Cell Signaling Technology, Danvers, MA, USA), anti-AKAP4 (Catalog No.: 611564; purified mouse anti-AKAP82 antibody; BD Biosciences, San Jose, CA, USA); anti-GSK-3α (1:1000 dilution; Ab21; Sigma-Aldrich, St. Louis, MO, USA); anti GSK-3β (1:2000 dilution; Novusbio, Littleton, CO, USA); anti-beta actin, and anti-alpha tubulin (1:10,000 dilution). Following brief incubation with TBST, the blots were incubated with the appropriate secondary antibody. The targeted proteins were then detected by enhanced chemiluminescence (ECL).

## Supplementary Materials

Supplementary Materials can be found at https://www.mdpi.com/1422-0067/21/19/7283/s1.

## Abbreviations

IPA	Ingenuity pathway analysis
Con	Control
Cap 60	Capacitation for 60 min
Cap 90	Capacitation for 90 min
