# Supplementary material for: Phosphoproteomics and Bioinformatics Analyses Reveal Key Roles of GSK-3 and AKAP4 in Mouse Sperm Capacitation
Source: Int J Mol Sci. 2020 Oct 2;21(19):7283. doi: 10.3390/ijms21197283 (PMC7582274; doi:10.3390/ijms21197283)
Supplement: Supplementary file 1 [file ijms-21-07283-s001.zip › Supplementary Files_IJMS/Supplementary Data IJMS.pdf]

Supplementary Data 1. GO analysis of the sperm capacitation phosphoproteome within the cellular component categories

Term	Frequency	Percentage
cytoplasm	572	15.38
membrane	401	10.78
nucleus	364	9.78
protein-containing complex	201	5.40
cytoskeleton	167	4.49
vesicle	121	3.25
mitochondrion	120	3.23
cilium	112	3.01
unclassified	93	2.50
others	1569	42.18

Details of the others

Term	Frequency	Percentage
intracellular organelle	109	2.93
cellular_component	107	2.88
neuron projection	107	2.88
synapse	102	2.74
endoplasmic reticulum	80	2.15
extracellular region	73	1.96
Golgi apparatus	65	1.75
cell part	60	1.61
chromosome	53	1.42
nucleolus	52	1.40
centrosome	51	1.37
cell projection	50	1.34
neuronal cell body	49	1.32
intracellular non-membrane-bounded organelle	45	1.21
intracellular membrane-bounded organelle	40	1.08
cell surface	40	1.08
cell leading edge	35	0.94
endosome	30	0.81
ribonucleoprotein complex	28	0.75
axoneme	28	0.75
vacuole	26	0.70
apical part of cell	25	0.67
site of polarized growth	21	0.56

extracellular matrix	19	0.51
receptor complex	16	0.43
kinetochore	15	0.40
filopodium	14	0.38
ubiquitin ligase complex	13	0.35
midbody	12	0.32
transcription factor complex	12	0.32
ribosome	11	0.30
spliceosomal complex	11	0.30
small ribosomal subunit	9	0.24
proteasome complex	9	0.24
brush border	9	0.24
nuclear pore	9	0.24
nucleosome	8	0.22
microvillus	7	0.19
organelle	6	0.16
microbody	6	0.16
polysome	6	0.16
RNA polymerase II, holoenzyme	6	0.16
transcriptional repressor complex	5	0.13
extracellular organelle	5	0.13
intraciliary transport particle	5	0.13
proton-transporting two-sector ATPase complex	4	0.11
photoreceptor inner segment	4	0.11
telomerase holoenzyme complex	4	0.11
septin complex	4	0.11
cell division site	4	0.11
transcription elongation factor complex	3	0.08
voltage-gated calcium channel complex	3	0.08
small nuclear ribonucleoprotein complex	3	0.08
stereocilium bundle	3	0.08
histone methyltransferase complex	3	0.08
cyclin-dependent protein kinase holoenzyme complex	3	0.08
PcG protein complex	3	0.08
protein serine/threonine phosphatase complex	2	0.05
AP-type membrane coat adaptor complex	2	0.05
F-actin capping protein complex	2	0.05
mRNA cleavage factor complex	2	0.05
preribosome	2	0.05

radial spoke	2	0.05
pyruvate dehydrogenase complex	2	0.05
retromer complex	2	0.05
cohesin complex	1	0.03
bleb	1	0.03
tricarboxylic acid cycle enzyme complex	1	0.03
death-inducing signaling complex	1	0.03
endopeptidase Clp complex	1	0.03
THO complex	1	0.03
exosome (RNase complex)	1	0.03
protein-DNA complex	1	0.03
endoplasmic reticulum Sec complex	1	0.03
basal part of cell	1	0.03
transcription factor TFIIH core complex	1	0.03
ubiquitin conjugating enzyme complex	1	0.03
BLOC complex	1	0.03
protein acetyltransferase complex	1	0.03
platelet dense tubular network	1	0.03
oxoglutarate dehydrogenase complex	1	0.03
condensin complex	1	0.03
transcription export complex	1	0.03
phosphatidylinositol 3-kinase complex	1	0.03
small nucleolar ribonucleoprotein complex	1	0.03
NADH dehydrogenase complex	1	0.03
translocon complex	1	0.03
respiratory chain complex III	1	0.03

Supplementary Data 2. 1050 Novel Phosphosites of Serine (S), Threonine (T), and Tyrosine (Y) Phosphorylation Corresponding to 402 Proteins

ID	Protein Name	Phosphopeptides	Novel Phospho sites (S/T/Y)
Q64441	1,25-dihydroxyvitamin D(3) 24-hydroxylase, mitochondrial	AAIKTMMSTFGKMMVTPVELHKRLNTKVWQA	T255*
		GKMMVTPVELHKRLNTKVWQAHTLAWDTIFK	T265*
Q8BHA9	2-phosphoxylose phosphatase 1	STGKSRTLQSGLALLYGFLPEFDWKKVYFKH	Y219*
Q9D8W5	26S proteasome non-ATPase regulatory subunit 12	APFDNEQSDLVHRISSDKKLEEIPKYKDLLK	S292*
Q8BJY1	26S proteasome non-ATPase regulatory subunit 5	FERVLMRVGYQAKNASTELKIRCLDAVSSLL	S354*
		FERVLMRVGYQAKNASTELKIRCLDAVSSLL	T355*
Q9Z2A0	3-phosphoinositide-dependent protein kinase 1	TAKVLSPESKQARANSFVGTAQYVSPELLTE	S244*
Q8JZS9	39S ribosomal protein L48, mitochondrial	VHRLCNRLSIKVEESYAMPTKTMEVMRLPDQ	Y124*
Q9CUX1	52 kDa repressor of the inhibitor of the protein kinase	INFDIKHDLDLMVDTYIKLYTNKSELPTINS	Y737*
P59384	A disintegrin and metalloproteinase with thrombospondin motifs 15	DIRQRGYKGLIGDDNYLALKNSQGKYLLNGH	Y752*
O88987	A-kinase anchor protein 3	______MADRVDWLQSQSGVCKVGVYSPGDN	S10*
		____MADRVDWLQSQSGVCKVGVYSPGDNQH	S12*
		TKEGKCPDDKPGTKKSFFYKEVFESRNAGDA	S239*
		VHQSLYMGDEPTPHKSLSTVASELVNETVTA	S171*
		STVASELVNETVTACSKNISSDKAPGSGDRA	S188*
		RARDKSESYSLISTKSRAGDPKLSNLNFAMK	S413*
		DPVRVLSWLRKDLEKSTAGFQDSRFKPGESS	S52*
		WLRKDLEKSTAGFQDSRFKPGESSFVEEVAY	S59*
		SPGRLHFEMSHKENPSQGLISHVGNGGSIDE	S109*
		ENPSQGLISHVGNGGSIDEVSFYANRLTNLV	S121*
		APKFCEDEEATGGALSGLTKMVANQLDNCMN	S662*
		KSTAGFQDSRFKPGESSFVEEVAYPVDQRKG	S66*
		STAGFQDSRFKPGESSFVEEVAYPVDQRKGF	S67*
		RKGFCVDYYNTTNKGSPGRLHFEMSHKENPS	S94*
		ATAVEKGRSVGEVLQSVLRYEKERQLDEAVG	S834*
		HLMDSVMKLCLIIAKSCDSPLSELGEEKCGD	S698*
		NFAMKSESKLKENLFSTCKLEKEKTCAETLG	S439*
		AAASTNFPTNKPPPPSPVVQDECKLKSPPHK	S565*
		NLLSETIFKSSRNCESNVHEQNTQEEEIHPC	S617*
		SELVNETVTACSKNISSDKAPGSGDRASGSS	S192*
		ELVNETVTACSKNISSDKAPGSGDRASGSSQ	S193*
		VTACSKNISSDKAPGSGDRASGSSQAPGLRY	S199*
		KNISSDKAPGSGDRASGSSQAPGLRYTSTLK	S204*
		ENKCVHQSLYMGDEPTPHKSLSTVASELVNE	T167*
		CEIPKVIVSNHNLADTVQNKQLQAVLQWVAA	T778*
		FPDNLYECLPVKGTGTAEALLQNAYLTIHNE	T736*

		GGKMDAQSFLEAAASTNFPTNKPPPPSPVVQ	T554*
		DAQSFLEAAASTNFPTNKPPPPSPVVQDECK	T558*
		FKSSRNCESNVHEQNTQEEEIHPCERPKTPC	T624*
Q60662	A-kinase anchor protein 4	AGTNDPKCKNQSLEFSAMKAEMKGKDKCKSK	S467*
		YMSQSAQYEKCGGGQSSKSLSMKHFETRGAP	S594*
		MSQSAQYEKCGGGQSSKSLSMKHFETRGAPG	S595*
		QSAQYEKCGGGQSSKSLSMKHFETRGAPGPS	S597*
		AQYEKCGGGQSSKSLSMKHFETRGAPGPSTC	S599*
		YSETPGPEVIVNNQCSTTNLQKQLQAVLQWI	S761*
		CLHHSMYTSGDKGKTSPRSAVSKIASEMAHE	S261*
		IQMARKEIKDKLEGGSKCLHHSMYTSGDKGK	S244*
		NKNKCGEKQQMCPKDSKEFADSISKGLMVYA	S321*
		EKEIIVIKDTEKQDQSKTEGSVCLFKQAPSD	S91*
		NKVCCTSKDEKREKISPSTDSLAKDLIVSAL	S536*
		GAPGPSTCMKENQLESQKMDMSNMVLSLIQK	S622*
		STLNVEDKDSKGAAGSRSEGELNLETLEEKE	S63*
		SLSMKHFETRGAPGPSTCMKENQLESQKMDM	S612*
		DWLHSRRGVCKVDLYSPKGQQDQDRKVICFV	S30*
		SKIASEMAHEAVELTSSEMRGNGEDCRDGRK	S282*
		KHKVGDLEGDYSKIPSENCYSVYADQVNFDY	S152*
		DLEGDYSKIPSENCYSVYADQVNFDYLNKGP	S157*
		GQQDQDRKVICFVDVSTLNVEDKDSKGAAGS	S48*
		LVESVMKLCLIMAKYSNNGAALAELEEQAAL	S714*
		GAALAELEEQAALVGSGSRCGRDAMMSQNYS	S732*
		ALAELEEQAALVGSGSRCGRDAMMSQNYSET	S734*
		KNTNNNQSPSNPATKSPSNQRSVATPEGECS	S198*
		MDMSNMVLSLIQKLLSESPFSCDELTESDNK	S640*
		MSNMVLSLIQKLLSESPFSCDELTESDNKRC	S642*
		MVLSLIQKLLSESPFSCDELTESDNKRCCDP	S645*
		GECSMDDLSFYVNRLSSLVIQMARKEIKDKL	S225*
		CMKNLHNITGVLMTDSDFVSAVKRNLFNHGK	S400*
		RSVATPEGECSMDDLSFYVNRLSSLVIQMAR	S218*
		RPEEQCQDNAELDFISGMKQMNRQFIDQLVE	S686*
		ASDMMVSVMKTLKVHSCGKPIPACVVLKRVL	S355*
		DLQKYALGFQHALSPSASSCKHKVGDLEGDY	S132*
		KYALGFQHALSPSASSCKHKVGDLEGDYSKI	S135*
		SRCGRDAMMSQNYSETPGPEVIVNNQCSTTN	T749*
		DKLEGGSKCLHHSMYTSGDKGKTSPRSAVSK	T253*
		KCLHHSMYTSGDKGKTSPRSAVSKIASEMAH	T260*
		EIIVIKDTEKQDQSKTEGSVCLFKQAPSDPI	T93*
		KGAAGSRSEGELNLETLEEKEIIVIKDTEKQ	T73*
		LSMKHFETRGAPGPSTCMKENQLESQKMDMS	T613*

		VSKIASEMAHEAVELTSSEMRGNGEDCRDGR	T281*
		QQDQDRKVICFVDVSTLNVEDKDSKGAAGSR	T49*
		LEKLPEVSAKAAEKGYSVGDLLQEVMKFAKE	Y811*
		GDLEGDYSKIPSENCYSVYADQVNFDYLNKG	Y156*
Q80YU0	Abhydrolase domain-containing protein 16B	ALGGDTLTRPPRGARSQVQCLLQQIRELPSQ	S82*
Q8CJ27	Abnormal spindle-like microcephaly-associated protein homolog	VRFLNLKKMVIKLQAHIRKYQQLQKYKKIKK	Y1564*
Q8K298	Actin-binding protein anillin	LGAVRTSNFTLVGSHTLSLSSVGDTKFALDK	T943*
Q9QY84	Actin-like protein 7A	TTYLMNLMNNSGKHFSEDHLGIVEDIKTRCC	S270*
		IAPEEHAVLVSDPPLSPHTNREKYAEMLFET	S180*
		______________MSLDGVWAPQTANIGDG	S2*
Q8BXF8	Actin-related protein T3	VYPNILGRSKGHTADSRQELCVGDQAQERRS	S45*
P56376	Acylphosphatase-1	RANFNNEKVIANLDYSDFQIVK_________	S93*
P08030	Adenine phosphoribosyltransferase	RASIRLLASHLKSTHSGKIDYIAGLDSRGFL	S55*
Q60614	Adenosine receptor A2b	RNRDFRYSFHKIISRYVLCQAETKGGSGQAG	Y308*
Q91WF3	Adenylate cyclase type 4	RVHITGATLALLAGAYAVERADTEHRDPYLR	Y422*
P61213	ADP-ribosylation factor-like protein 4A	PVLIVANKQDLRNSLSLSEIEKLLAMGELSS	S143*
Q3V132	ADP/ATP translocase 4;ADP/ATP translocase 4, N-terminally processed	APIERVKLLLQVQASSKQISPEARYKGMLDC	S55*
Q9CXB8	Alpha-protein kinase 1	ICHRLSLTRPSLEQTSKV_____________	S1224*
		NQHASCNEICHRLSLTRPSLEQTSKV_____	S1229*
		ASCNEICHRLSLTRPSLEQTSKV________	T1221*
Q8K4E0	Alstrom syndrome protein 1 homolog	QAWNLKFNLGRDRGYSISELNEDDRRKVEEI	S1684*
		SADQKIGAAVVSSAYSQEIKPGSFHQEELPD	S430*
		PDDQKIPLPTTFHGSSDQKVKPVIFVQKQLR	S1257*
		PGNTLSAMSFKVPRISGSTEQTNVTTGSSSS	S891*
		NTLSAMSFKVPRISGSTEQTNVTTGSSSSYS	S893*
		VSFIRSKKVVCFKESSTTDVCTQRESFVEEV	S1470*
		LRSPQGIGCLPEAVCSRIIIESHEKGCFRTL	S1731*
		VPGPTDQKTGIATVHSTSQSYIGRRTVSYQK	S992*
		GPTDQKTGIATVHSTSQSYIGRRTVSYQKEF	S994*
		TVHSTSQSYIGRRTVSYQKEFPDLSEKALKV	S1004*
		GTTGTVDQKTVTPTMSSSFLQKEKPSIFYQQ	S764*
		TGTVDQKTVTPTMSSSFLQKEKPSIFYQQTL	S766*
		ETGVRWGSEEDQRTESWHCLPQERDSSQTLA	S173*
		PGPTDQKTGIATVHSTSQSYIGRRTVSYQKE	T993*
G3UZ78	Androglobin	LDRDLISLTTATLDKSQEELAINEGVAKEIW	S616*
		FIKKPVTTDTTTSAPSPETLSVSQSQTKSSE	S1431*
		TTTSAPSPETLSVSQSQTKSSEEGELDTGKY	S1440*
		LKEILPEFKLTEEPSSESKITTIDNKLKEAT	S306*
		KEEPPQKEIPVVRQRSPTILETSPQQIRKAL	S1509*
		TFVSGARWKLRLIGSYNPLPFLARDSPCNTF	Y1087*
Q9D2J7		FTKKVDITFIPKRIWSPEATTAELIRKRELR	S726*

	Ankyrin repeat and EF-hand domain-containing protein 1	ATSEQLLSVAQMHEKSRGGGVNINEFFKGTK	S385*
Q91ZT8	Ankyrin repeat and SOCS box protein 9	LRIRKCFGIRQHHKISELLLPEDLKRFLLHL	S275*
		TIDWRTPLFNACVSGSQDCVNLLLQHGATPH	S110*
Q810N6	Ankyrin repeat domain-containing protein 45	RLKNEWLESHPEASISEIFEQKQQLEDIVSP	S219*
Q8VE42	Ankyrin repeat domain-containing protein 49	TRDEDEYTPLHRAAYSGHIDVVRELVAKGAD	S117*
Q9CQM6	Ankyrin repeat domain-containing protein 61	RSSNIRDRSLLARLLYRTYPLRLSNKQGILP	Y335*
		NIRDRSLLARLLYRTYPLRLSNKQGILPAGI	Y338*
Q8C5H1	Anoctamin-4	IVFEHLVFCIKHLISYLIPDLPKDLRDRMRR	Y904*
Q75UR0	Anoctamin-5	NLFLRRRLMFQRSEHSKDSVFFRDGIRQIDF	S59*
E9Q414	Apolipoprotein B-100;Apolipoprotein B-48	TFLDSMCTSTLQFLEYALKVVETHKIEEDLL	Y3891*
Q9CPT0	Apoptosis facilitator Bcl-2-like protein 14	TRSLSQKALGTWSTDSWTQVSLPCRGSPSSE	S65*
		DVIHSQGGSKLKERVSEILYFRFEGPCDSKN	S187*
		EDDDPNSIEFKILAFYARHHVFKNTPAVFSP	Y30*
Q80X86	Armadillo repeat-containing protein 12	YKAVRTGLKCHPPLCSNSPICIARLAIERER	S49*
		AVRTGLKCHPPLCSNSPICIARLAIERERHG	S87*
		RLLNSLDCKQDEYTRSMILHNITRCVYLLEA	S327*
		VIVSLQCPQDLGSRPSSCRPSHSCFKTGK__	S332*
A2AU72	Armadillo repeat-containing protein 3	SLRRGSKEKANAIFGSPTEEKSEPASVRNTI	S548*
B2RY50	Armadillo repeat-containing protein 4	MVNILDSPHKSLKCLSAETIANVAKFKRARR	S308*
		VTCLKDKSPVFSENMSKHEIRFTEEQQKDNQ	S714*
		TRDLVRLHGGLKPLASLLNNTDNKERLAAVT	S512*
P47857	ATP-dependent 6-phosphofructokinase, muscle type	PILKILAKYEIDLDTSDHAHLEHISRKRSGE	S762*
Q9WUA3	ATP-dependent 6-phosphofructokinase, platelet type	MKILAKYEASYDMSDSGKLESLQHHEEL___	S772*
Q6P9R1	ATP-dependent RNA helicase DDX51	HHEIPRKLLQPLVARYETALSQLEKTVKEEQ	Y619*
Q8BVN8	Axonemal dynein light intermediate polypeptide 1	GPSSTSPQPPKAKLPSTSCVPDPTKQAEEIL	S54*
		RPLKVTIQQPGPSSTSPQPPKAKLPSTSCVP	S44*
Q80YW5	B box and SPRY domain-containing protein	FERAEEAEGILDPQDSEKLSFNEKCAWSPLL	S247*
		LISIVRGPLATVRLDSPPHHPCRSGHASR__	S460*
O88870	Bestrophin-1	HMKKKTVEFNLNIPESPTEHLQQRRLDQMST	S509*
P15327	Bisphosphoglycerate mutase	YKVCDVPLDQLPRSESLKDVLERLLPYWKER	S157*
E9Q173	BTB/POZ domain-containing protein 16	SFSMQRIKHTDLECPSPVCEHSTISLRSERL	S448*
Q52KB6	C2 domain-containing protein 3	GLVPGGGVQFEVWCRYYYPNVRDQMVAKGTL	Y1100*
		LVPGGGVQFEVWCRYYYPNVRDQMVAKGTLP	Y1101*
		VPGGGVQFEVWCRYYYPNVRDQMVAKGTLPL	Y1102*
Q8VHF2	Cadherin-related family member 5	TVQAGDSPSAVRSILTKERRPEGEGGYKAVW	T762*
Q63811	Calcineurin subunit B type 2	IRRLGKSFRKLDLDKSGSLSIEEFMRLPELQ	S35*
Q9D424	Calcium-binding tyrosine phosphorylation-regulated protein	VVVGPVSEAEPPKASSAPLQGEQEPPAHEAP	S257*
		EQEPPAHEAPDTQVTSASRISSIYNDVPVNE	S278*
		SNKTTQFPSVHAEVQSEETSEGARGPSDKPT	S131*
		PLQGEQEPPAHEAPDTQVTSASRISSIYNDV	T274*

		KAVEPKTKISIESLKTVQVEENSQHKSSVHV	T345*
Q8CDE2	Calicin	DLAYSGIRDNFHFWASPEGSMHFMRCPPVIF	S165*
		LIESVLMDRKQERPCSLLSYQRKGALLDSVV	S267*
		TLMFASNKLVGLENNSAHATLIESVLMDRKQ	S247*
		VDQLLDYFYSGKVVISEQNVEELLRGAQYFN	S97*
Q9DBC7	cAMP-dependent protein kinase type I-alpha regulatory subunit;cAMP-dependent protein kinase type I-alpha regulatory subunit, N-terminally processed	RNISHYEEQLVKMFGSNLDLMDPGQ______	S392*
		ADLEVPVPSKFTRRVSVCAETFNPDEEEEDN	S96*
		KDNQTRSVGQYDNRGSFGELALMYNTPRAAT	S202*
		KGIGFAITRDLCRKFSGDVVLAARDEERGQT	S30*
Q8BK63	Casein kinase I isoform alpha	___________MASSSGSKAEFIVGGKYKLV	S5*
Q8BVP5	Casein kinase I isoform gamma-2	PENFLVGRPGSKRQHSIHIIDFGLAKEYIDP	S183*
O54833	Casein kinase II subunit alpha'	GPAAGSRARVYAEVNSLRSREYWDYEAHVPS	S18*
		AGSRARVYAEVNSLRSREYWDYEAHVPSWGN	S21*
Q91ZR5	Cation channel sperm-associated protein 1	FHQQNELQHLREFSDSHDNAFSHHSYQQDRA	S78*
		SQIFGKAQSRESLRESASLSEGEDHVQKRKK	S297*
		IFGKAQSRESLRESASLSEGEDHVQKRKKAQ	S299*
		GKAQSRESLRESASLSEGEDHVQKRKKAQRA	S301*
		KLEQAARVHEKLLDDSLTDLNKADANAQMTE	S607*
Q80W99	Cation channel sperm-associated protein 3	__MSQHFHHNPVRVKSGSLFATASEALQARL	S14*
		MSQHFHHNPVRVKSGSLFATASEALQARLSK	S16*
P28033	CCAAT/enhancer-binding protein beta	RKSRDKAKMRNLETQHKVLELTAENERLQKK	S252*
Q62192	CD180 antigen	SSIDFIPLHNQKTLESLYLGSNHISSIKLPK	S153*
Q9CXS4	Centromere protein V	AAEEPPPAVTPAASVSALDLGEQRERWETFQ	S84*
Q8BI22	Centrosomal protein of 128 kDa	SLELERAVAELENVKSESELLKKQLTNERQT	S1014*
		VERMRLEHGIKRRDKSPSRLDTFLKGIEEER	S439*
B2RX88	Centrosome and spindle pole associated protein 1	KKKRREKDLAFGITTSGVQDPEKSPDRLKQF	S397*
		VPEEPNTQISAAENKSVHCNGPPRSADLDIT	S331*
Q03311	Cholinesterase	DSLITRKEFQEGLNMYFPGVSRLGKEAVLFY	Y386*
B9EKE5	Ciliogenesis-associated TTC17-interacting protein	IITKMPLIREEDVIESPPTFDKKPLVWGEDL	S269*
Q9D4C9	Clavesin-1	DWSNFSFKQASKLTPSILKLAIEGLQDSFPA	S200*
Q8R3P7	Clusterin-associated protein 1	KAARQLASEITAKGASLYDLLGKEVELRELR	S153*
		LAQVQKTKDLLNNVASDEANLEAKIEKRKLE	S208*
P70375	Coagulation factor VII;Factor VII light chain;Factor VII heavy chain	CAAIGHIGVYTRVSQYIDWLVRHMDSKLQVG	Y424*
Q9D4K7	Coiled-coil domain-containing protein 105	MNQPLDKVLEQAGRHSWVDITRPPTPRTQGL	S264*
		AQRLVNDWDPPTPARSQTNTASK________	S492*
		DKVLEQAGRHSWVDITRPPTPRTQGLKTPPP	T268*
		EQAGRHSWVDITRPPTPRTQGLKTPPPDPIG	T272*
		CYEQAQRLVNDWDPPTPARSQTNTASK____	T488*
		VDITRPPTPRTQGLKTPPPDPIGTYTPACAK	T280*
Q8C5T8	Coiled-coil domain-containing protein 113	MSLKGYRKAWNKMKTSDEQLQIIRPPGK___	S365*
Q80X53		RAGLEVLEQPLKGRGSFTHHF__________	S536*

	Coiled-coil domain-containing protein 116	________MARCRHHSGYLADDEAAHSTYVA	S8*
		CRHHSGYLADDEAAHSTYVAPLPKKHLLPEM	S19*
		SHACRHNGFPLFPVTSETIPDLPGNSDLLQP	S340*
		AGVPLVDIQDPVEVPSSRHRSRARPSIDTVH	S117*
		GVPLVDIQDPVEVPSSRHRSRARPSIDTVHR	S118*
		EMRPTCKLGRVPHLPSMNQYSEHQSHQQNFR	S48*
		FLAKHRLFPALQRVVSQAVDKLSHACRHNGF	S318*
		FPLFPPFLSLGKSFSTSPPTLHPEVTSRAGL	S509*
Q3TVA9	Coiled-coil domain-containing protein 136	EMEIASLRAEMELKTSEPSNLSISDFSGIQD	S206*
		AEMELKTSEPSNLSISDFSGIQDELHHLRER	S214*
		ELKTSEPSNLSISDFSGIQDELHHLRERYNL	S217*
		KVLTKLQAVKALYQVSQEEHCQLQQRMHRLL	S856*
		LEVQHHLHDKLRCHESEVHRLRSMVDCLREK	S453*
		HDKLRCHESEVHRLRSMVDCLREKNEKNSGI	S460*
		TELQQLRLLRCTPVESQSEKELMCRLQKLQA	S410*
		LQQLRLLRCTPVESQSEKELMCRLQKLQAQH	S412*
		LRSMVDCLREKNEKNSGIHLQLQEMKGLYQF	S473*
		ERYNLLNEEYQALRESNSSLTGQLAELESDR	S243*
		YNLLNEEYQALRESNSSLTGQLAELESDRTR	S245*
		NLLNEEYQALRESNSSLTGQLAELESDRTRR	S246*
		DDMERIRGDYEMEIASLRAEMELKTSEPSNL	S196*
		GIHLQLQEMKGLYQFSRDELERQKHMYDQLE	S498*
		EKSYCSSSTSVSYKKSYGSVSSGETLHRSYA	S812*
		YCSSSTSVSYKKSYGSVSSGETLHRSYASSS	S815*
		SSTSVSYKKSYGSVSSGETLHRSYASSSTDE	S818*
		ELMCRLQKLQAQHQCSVNEKEQLLEVQHHLH	S430*
		LAQAEIQNLRQAAADSATEHESDIASLQDDL	S158*
		QNLRQAAADSATEHESDIASLQDDLCRLQND	S164*
		QAAADSATEHESDIASLQDDLCRLQNDLDDM	S168*
		QCCQEELRQLKENRPSISSEARGKNVNKNMN	S968*
		CQEELRQLKENRPSISSEARGKNVNKNMNKN	S970*
		QSPMIKRSFEYCGKNSGSRAPSTENFHRSYE	S744*
		FLLCQQELTELKSSQSLCEENGNCSNKCDAL	S522*
		YGSVSSGETLHRSYASSSTDEDPAEPEDLEH	S828*
		GSVSSGETLHRSYASSSTDEDPAEPEDLEHF	S829*
		SVSSGETLHRSYASSSTDEDPAEPEDLEHFE	S830*
		VSSQPSTETFLKSYDSSTSANEAFEKSYCSS	S788*
		ESSIDENEGYQKSYVSSQPSTETFLKSYDSS	S774*
		DENEGYQKSYVSSQPSTETFLKSYDSSTSAN	S778*
		ELKSRLCTLQQKYDASQDEHSELLKVQMQLE	S379*
		LCTLQQKYDASQDEHSELLKVQMQLETELQQ	S384*
		MQLETELQQLRLLRCTPVESQSEKELMCRLQ	T406*

		LNEEYQALRESNSSLTGQLAELESDRTRRAT	T248*
		QAEIQNLRQAAADSATEHESDIASLQDDLCR	T160*
		ENEGYQKSYVSSQPSTETFLKSYDSSTSANE	T779*
Q8BSN3	Coiled-coil domain-containing protein 151	ERMERKTHRDHVLLQSEDTIQDHQRHREEEL	S327*
Q8C963	Coiled-coil domain-containing protein 159	THWSRVLEPEIVNTASEQAGKSGAWEKEWDS	S83*
A0JLY1	Coiled-coil domain-containing protein 173	RQCQFHQTERVKRFHSGLLLSRVMKERDVQI	S151*
Q8CDV0	Coiled-coil domain-containing protein 178	DVNKAIYFSYPSRRHSCTLVNIPKPCVNKMI	S86*
A2AJB1	Coiled-coil domain-containing protein 183	IQYQTKVTTLVERVKSAVQCSHLWDIAGRFL	S308*
Q3V036	Coiled-coil domain-containing protein 27	KELHVEPGSAIEEKSSEGPPEEAAAAKLSRP	S309*
		SEKKPQSTQQSLKKSSQAAAGYFYDKEDQIR	S52*
		GSASSVDGRVFPFSKSACELNYPRKRSEPSD	S148*
Q8CDN8	Coiled-coil domain-containing protein 38	SHLGQLAFFADAGSSSYERLGLDPTLILRLT	S85*
		KKKLGRRLIYRSKPQSADKQELLLVSDTRSK	S539*
		HEFINDQRDRFLLEYTVSTKKKTIKRFERLI	T125*
Q8CDV6	Coiled-coil domain-containing protein 63	NFLQRERTKELQDTMSEKGDEIRLKKKVIG_	S544*
Q9D5W4	Coiled-coil domain-containing protein 81	SSPGCQRNDNERPRTSPAPACQDHNKAGQEM	S311*
		REISSPKKHRERQSISPAKVTSVSLLDKFER	S267*
		AMAFPRIEHKETENKTPVEVVGEEGGENRPR	T222*
Q80X19	Collagen alpha-1(XIV) chain	QNYTVQLIAYYKDKESKPAQGQFRIKDLEKR	S108*
Q8C0L8	Conserved oligomeric Golgi complex subunit 5	PEKALKDSLQPYEAAYLSKSLSRLFDPINLV	Y454*
Q6A0A9	Constitutive coactivator of PPAR-gamma-like protein 1	SAHALKLSRNGKSLTTSQYLMHEVAKQLDLN	T214*
Q9CZ04	COP9 signalosome complex subunit 7a	SQDPEQHLTELREPASGTNQRQPSKKASKGK	S247*
Q8VBV7	COP9 signalosome complex subunit 8	EEAVKGVLEQGWQADSTTRMVLPRKPASGTL	S157*
Q9D8X1	Copper homeostasis protein cutC homolog	KFRNSSVAMGASLAHSEYSLKVTDVTKVRTL	S248*
Q80T79	CUB and sushi domain-containing protein 3	SGVYKSQEARLMLHIYLIKVPAHASVKKMKE	Y3557*
Q9D4H8	Cullin-2	YAETKIFLESHVRHLYKRVLESEEQVLVMYH	Y73*
Q9D5U8	Cyclic nucleotide-binding domain-containing protein 2	SQKIKSWTAKKKRKLYQLVIDIIMMNRVCKM	Y110*
Q3TVC7	Cyclin-D1-binding protein 1	ICEQVRIAIEEIIVYYSLPKDQGITLRKLV	Y100*
		CEQVRIAIEEIIVYYSLPKDQGITLRKLVR	Y101*
P49615	Cyclin-dependent-like kinase 5	GIPVRCYSAEVVTLWYRPPDVLFGAKLYSTS	Y167*
Q91YE9	Cytosolic 5'-nucleotidase 1B	RPRPPKPKHAITIAVSSRALFNMVDDRKIYE	S274*
		IRPHIFFDDQMFHIESAQKFGTITAHVPYGI	S553*
		TEWKPYAQRRLQYGTSVDMEPEYISDGPQQR	S214*
		RLQYGTSVDMEPEYISDGPQQRQRQQTEEDE	S223*
		TEPNSRRTSAKMYTGSDPWAHRENREPRDLQ	S158*
		LHNTDSRGYVVRNQWSRTSRSPSTGAPSVDE	S63*
		SRGYVVRNQWSRTSRSPSTGAPSVDEPRSRN	S68*
		GYVVRNQWSRTSRSPSTGAPSVDEPRSRNTA	S70*
Q8VDR9	Dedicator of cytokinesis protein 6	RGSGRPASLYLARSKSISSSNPDLAVVPGSV	S878*

		KSISSSNPDLAVVPGSVDDEVSRILASKGVD	S892*
		NPDLAVVPGSVDDEVSRILASKGVDRSHSWV	S898*
E9Q557	Desmoplakin	LVFDGLRKKVTAMQLYECQLIDKTTLDKLLK	Y1993*
O88673	Diacylglycerol kinase alpha	KRPHGDTCEINQALGSAAKIITDPDILKTCV	S626*
Q91WG7	Diacylglycerol kinase gamma	YEGGSLTKILKEIEQSPLVMLDRWYLEVMPR	S547*
O09035	Diazepam-binding inhibitor-like 5	______________MSQVEFEMACASLKQLK	S2*
		____MSQVEFEMACASLKQLKGPVSDQEKLL	S12*
P98192	Dihydroxyacetone phosphate acyltransferase	PSSSSSRFSVGSASPSSVLLYAKDLKKWDEF	S19*
Q811Q4	Disintegrin and metalloproteinase domain-containing protein 29	LPNWISYSLKLGGQRYIIHMKIKNLFLTRHL	Y72*
Q9CWV1	DNA helicase MCM8	HPRLSTDAAQALQDFYLELRKQSQRVGSSPI	Y686*
D3YU81	DNA-binding protein RFX8	IGLLKSDLRAIIREGTLDISKKVLEGDSDHE	T332*
P60898	DNA-directed RNA polymerase II subunit RPB9	GFVGIRFCQECNNMLYPKEDKENRILLYACR	Y25*
O54946	DnaJ homolog subfamily B member 6	RPQKPPRPAPTAKLGSKSNWEDDEQDRQRVP	S313*
		QKPPRPAPTAKLGSKSNWEDDEQDRQRVPGN	S315*
P60904	DnaJ homolog subfamily C member 5	__MACNAPNQRQRTLSTSGESLYEILGLHKG	S14*
		MACNAPNQRQRTLSTSGESLYEILGLHKGAS	S16*
		NAPNQRQRTLSTSGESLYEILGLHKGASCEE	S19*
		SGESLYEILGLHKGASCEEIKKTYRKLALRH	S31*
		_MACNAPNQRQRTLSTSGESLYEILGLHKGA	T15*
Q3TDQ1	Dolichyl-diphosphooligosaccharide--protein glycosyltransferase subunit STT3B	YLGDDMKRENPPVEDSSDEDDKRNPGNLYDK	S495*
		LGDDMKRENPPVEDSSDEDDKRNPGNLYDKA	S496*
Q69Z23	Dynein heavy chain 17, axonemal	LPIPEHLGSLDGTLDSMERIPSSMDNSLLHS	S174*
		KSGRNYGPPGTKKLIYFIDDMNMPEVDKYGT	Y2503*
		RMNSFFHRVQTIEDLYKTAIEFLKLEKIELG	Y428*
Q91XQ0	Dynein heavy chain 8, axonemal	DQPPASGKPLKKEERSFEETIPARKLKNFYP	S1200*
		GQARVRARHTPRPAQSVLSDGISQSSRRPSK	S122*
		HPEDPKLLSEQGPATSVTDYRSLIPSDEEVT	S84*
		TMKRRIFVGSQGRRRSEDIVSFIKTEVHLAI	S1118*
		DGISQSSRRPSKFRRSMTGIPNLQETLKEKQ	S141*
		KVGDKECDIMDTFKLYITTKLPNPAFTPEIN	Y3799*
Q8C0M8	Dynein intermediate chain 1, axonemal	PKEGEGEAEAEAEAGSQTDIPAAAETTEKVI	S159*
		QSISVTRGARRRDEDSGTDVGEGTDEWSQSK	S24*
		ISVTRGARRRDEDSGTDVGEGTDEWSQSKAT	T26*
		AVIKNADMSEEMQQDSVECATQALEKYNIEK	S21*
F2Z461	E3 ISG15--protein ligase Herc6	TEKWISSLITTCLEEYLLRDLPYSSPHQEAL	Y464*
Q7TMY8	E3 ubiquitin-protein ligase HUWE1	TMEKVVRSAATSGAGSTTSGVVSGSLGSREI	S1852*
Q80X56	E3 ubiquitin-protein ligase TRIM69	QLTLDPKTAHPNLVLSKSQTSVCHCDVKQVM	S340*
		DPKTAHPNLVLSKSQTSVCHCDVKQVMPDDP	T344*
Q80X60	EF-hand calcium-binding domain-containing protein 3	LKAPVFHKRDKDLRGSFSDHLKHKESKLSPA	S30*
		APVFHKRDKDLRGSFSDHLKHKESKLSPAQL	S32*
		IELVSYFRKRFQESHSEIMWSSYGRRGLKSE	S172*

		DWKTQAMNVKPLKSASGYSDDILAIDHLFKK	S294*
		TQAMNVKPLKSASGYSDDILAIDHLFKKKQH	S297*
A0JP43	EF-hand calcium-binding domain-containing protein 5	NMRQDVSIIPPLPKKSATSIYAISSERSIRE	S1398*
Q6P1E8	EF-hand calcium-binding domain-containing protein 6	EISWASKARPKEKEESSPPPISFSKVTPDEV	S1061*
		KARPKEKEESSPPPISFSKVTPDEVIKTMQE	S1067*
		LYSPPRDSRVGLKSRSHPCTPVGTPPLQNCE	S1311*
		FSQGTRSNLYSPPRDSRVGLKSRSHPCTPVG	S1303*
		SNLYSPPRDSRVGLKSRSHPCTPVGTPPLQN	S1309*
		MLSGLETVPLQSRTASRTNMDEHFISAEECL	S739*
		KQTFDPEGTGAVRVNSLLDVLDDSTKVRKMS	S487*
		EKEESSPPPISFSKVTPDEVIKTMQEVVESS	T1072*
		PRDSRVGLKSRSHPCTPVGTPPLQNCEPIES	T1315*
		RVGLKSRSHPCTPVGTPPLQNCEPIESRLRK	T1319*
Q8CDU5	EF-hand domain-containing family member B	QDRKWRERMGSSEAKSPPCHALGVGLERHTI	S83*
		_______MCSFVRVGSPKPLQTSASPLEMSS	S9*
Q9JK48	Endophilin-B1	TQKRIGTADRELIQTSALNFLTPLRNFIEGD	S139*
Q6SP97	Enkurin	_____________MDSPCTSESIYNLIPSDL	S3*
		_______MDSPCTSESIYNLIPSDLKEPPQH	S9*
Q9D9V2	Equatorin	AMNEIDAQLLLNKEGSPSN____________	S334*
Q8BFZ9	Erlin-2	FMDSAGGLGKQFEGLSDDKLGFGLEDEPLEA	S320*
Q9Z0N2	Eukaryotic translation initiation factor 2 subunit 3, Y-linked;Eukaryotic translation initiation factor 2 subunit 3, X-linked	RFKNELERNITIKLGYANAKIYKLDDSSCPR	Y83*
		ERNITIKLGYANAKIYKLDDSSCPRPECYRS	Y89*
Q6NZJ6	Eukaryotic translation initiation factor 4 gamma 1	RSQQGPRKETRKIISSVIMTEDIKLNKAEKA	S722*
Q8BGY2	Eukaryotic translation initiation factor 5A-2;Eukaryotic translation initiation factor 5A-1	GIDIFTGKKYEDICPSTHNMDVPNIKRNDYQ	S75*
P70190	F-actin-capping protein subunit alpha-3	SFRFDLLQNQLRDIQSHGIIRNETEYLRSVV	S106*
Q8BIG4	F-box only protein 28	LARHADILAAVETRLSLLNMTFMKYVDSNLC	S151*
		DILAAVETRLSLLNMTFMKYVDSNLCCFIPG	T156*
Q3TQF0	F-box only protein 31	WLELKSFSLYSRVQATFQNAAAPSPQAFDEM	T483*
Q5SUS0	F-box/WD repeat-containing protein 10	EFSKSRVHLKQTKNLSSDDMETPVGEVSHPL	S765*
Q8R123	FAD synthase;Molybdenum cofactor biosynthesis protein-like region;FAD synthase region	NTAVQFHLKELYVAASEGSIAPILSEAQAHF	S194*
Q5SXJ3	Fanconi anemia group J protein homolog	QGGEKTDFDELLQVYYDAIKFKGEKDGALLI	Y746*
Q9QXW4	Fascin-3	RPFLVLRGRYGYVGSSSDHDLLKCNMDQPDC	S401*
		HHFLSRVDRLVPQRSSQTAFHMQVRPRGLVA	S211*
O08716	Fatty acid-binding protein 9	FEETTADNRKVKSLITFEGGSMIQVQKWLGK	T86*
A1EGX6	Fibrous sheath CABYR-binding protein	PPAPKATHSIGNIPGSKDNYSRKEYESLRLS	S64*
		EQTPADQVLLKEHRLSQVADISEKELESTTL	S1020*
		THDVPTEVQVPQAKESPGQVLPLSGESTAEE	S757*
		VEAQLTSVEESPKRASVDVQPLSPETPVEES	S958*
		EYRRRRRPSQPMVDKSQQTEITEKRKAMASV	S32*

		KRKAMASVQPPAPKATHSIGNIPGSKDNYSR	T55*
A2ARZ3	Fibrous sheath-interacting protein 2	SACSKTANVAANKAASSTVAEDSQQCVDGRH	S21*
		ACSKTANVAANKAASSTVAEDSQQCVDGRHK	S22*
		KEVTKSKAKNDLPTSSKTEIPKEMCPQNLKN	S1560*
		SRSLTSDKALVILKDSAQLVPAKDLSSSGHR	S3414*
		SRSLTSDKALVILKDSAQLVPAKDLSSSGHR	S5816*
		ILKDSAQLVPAKDLSSSGHRDIAAKEKASKE	S5827*
		LKDSAQLVPAKDLSSSGHRDIAAKEKASKET	S5828*
		ALEESCQEPQEGNSNSLEFVTIYKGSKHIAG	S6925*
		NLKRFLALSKCCQPTSGENIESIEESGIQIM	S6816*
		ETCTKKSKDATTETESLEISLLYDKKAKAMD	S664*
		VKHILTDMLKDMSSDSSSHLGSQTGKGASAF	S1118*
		DMLKDMSSDSSSHLGSQTGKGASAFISEKSQ	S1124*
		KDTTSLTKPELNKIASQLTKSVTAEISKSNI	S6477*
		KVSASSVTYPAEVQNSSSEQKRSEVTKRLSD	S394*
		SASSVTYPAEVQNSSSEQKRSEVTKRLSDER	S396*
		KIVHSCVCNIFKECKSQESICENINSNGENL	S6027*
		HSCVCNIFKECKSQESICENINSNGENLAKR	S6030*
		DVISDIIGFSMVKEISNSEFQPQAEEELSSS	S6388*
		YDTAMRLVNSMTKELSDAEIKVFRPEKEDEV	S5932*
		EKEMQSMVDSIYADLSHSNLYQSLTKDKKGL	S4882*
		PTSPPEVKETVTKEPSPSTNIKNEDQMSDVQ	S5970*
		KEPSPSTNIKNEDQMSDVQKTPEQSSPDKLP	S5982*
		RGKNSTDDSARESIISTQLSPTRNAKLSQIS	S426*
		STDDSARESIISTQLSPTRNAKLSQISLDHQ	S430*
		KEDESKVKPATRETTSPPTSPPATMKSQGSQ	S5514*
		SKVKPATRETTSPPTSPPATMKSQGSQVQQL	S5518*
		AFLEDVIVDLAHKFCSFLTLTDDARKKDLPE	S5305*
		PYTVQLPYKFLEDVTSGLLAKVFSKLSNVKT	S6115*
		DVTRFQNWLLQDNTHSIKDQERLLRHRYLDM	S195*
		SPPTTMKSRGAQVQESVTSPFTTKESRGAQV	S5605*
		TTMKSRGAQVQESVTSPFTTKESRGAQVQQS	S5608*
		AQVQESVTSPFTTKESRGAQVQQSAMSPPPS	S5615*
		TTMKGKGAQVQQFATSPPTSMKSQGSQVQQS	S5672*
		SPFTTKESRGAQVQQSAMSPPPSMKDRGAQV	S5623*
		TTKESRGAQVQQSAMSPPPSMKDRGAQVQES	S5626*
		SIAELRRNSIRGIGPSSTDVSEMGTRQKERR	S6671*
		IAELRRNSIRGIGPSSTDVSEMGTRQKERRI	S6672*
		KVVNSVYTNLLKHCGSPTSAFEDLMRKSDVI	S5360*
		SEDIVKTKHCLGQEPSIFSQDQKHQIQKASE	S1085*
		IVKTKHCLGQEPSIFSQDQKHQIQKASENIV	S1088*
		TIYKGSKHIAGSARLSKEHVSEMPRSSISKQ	S6945*

		KLVGETVTRVLSKIFSPNTNINVELENVTQK	S4333*
		NYQKTSLDYQFPKIGSSKSDSHLLTSLETCT	S637*
		YQKTSLDYQFPKIGSSKSDSHLLTSLETCTK	S638*
		KTSLDYQFPKIGSSKSDSHLLTSLETCTKKS	S640*
		SLDYQFPKIGSSKSDSHLLTSLETCTKKSKD	S642*
		VVKIIDELKSQEKPASITDVMLDSRVLEEAL	S6431*
		SQETEMRKITSKILHSIQEFISKSKIKVVQP	S6313*
		ITSKILHSIQEFISKSKIKVVQPVKESEAVP	S6321*
		ILASEPTHYFIHRIMSTSSYNQEDLISGEAE	S6885*
		EQSSPDKLPALEKIPSIEKSIVNKIVHSCVC	S6004*
		SPPTSMKSQRIQVQQSVMSPPTTMKGKGAQV	S5551*
		TSMKSQRIQVQQSVMSPPTTMKGKGAQVQQF	S5554*
		LKKGGYITSNNKIVCSLRELNKYRQYLTSLK	S133*
		HLEKIVTQLMSQINPSDSSVGHSDTYQSELS	S4818*
		KIVTQLMSQINPSDSSVGHSDTYQSELSNTV	S4821*
		EPIHKVHQQTEQKKASFASDPANQIVKEEIQ	S868*
		HKVHQQTEQKKASFASDPANQIVKEEIQSLI	S871*
		PERTKSQETEMRKITSKILHSIQEFISKSKI	S6308*
		SDMPIKKPPTPLKPPSAHVERTVIDQTYQRQ	S598*
		NGGLSKKSSYAGEPGSPTDAPPQGIFSFPVN	S471*
		SDIKGTKKAFPKKVASLIVDGVSSVPSCRVI	S6549*
		KAFPKKVASLIVDGVSSVPSCRVITESSYTA	S6556*
		AFPKKVASLIVDGVSSVPSCRVITESSYTAA	S6557*
		KKVASLIVDGVSSVPSCRVITESSYTAAHGD	S6560*
		TGRLDVKPLEAVGRNSFQNVRRPDITRVELL	S6707*
		SDILNMVFAKLEHFASGSVVNVGNLNDENKK	S1593*
		ILNMVFAKLEHFASGSVVNVGNLNDENKKEH	S1595*
		QNKVGSHDWGLKLELSLSSEDIVKTKHCLGQ	S1067*
		VGSHDWGLKLELSLSSEDIVKTKHCLGQEPS	S1070*
		NISEKLFRPSYGFNLSDPYCRLLETQYKSLH	S86 *
		YHTLNAEIILYKLHNSLKNFNSEPMTSKDYS	S4131*
		QIPPDLVANLPLRLHSKLSQNVLVNKVHYDI	S4767*
		NLSDPYCRLLETQYKSLHDPHLRTYYKRKDI	S99*
		GEGSHSSKLPHFGGASVVAKSGKEKAPSSLF	S5272*
		LKLFVCPKLQIGNKSSLIEKSLHSQLSTYTM	S1278*
		SIISTQLSPTRNAKLSQISLDHQKEEKEMKS	S438*
		STQLSPTRNAKLSQISLDHQKEEKEMKSTWN	S441*
		EMLGKLTGDHPDPQPSCSHQNTETIDKDPPT	S1457*
		LGKLTGDHPDPQPSCSHQNTETIDKDPPTMS	S1459*
		SHQNTETIDKDPPTMSNTQLISKAILENVLA	S1474*
		QKPGELYDTAMRLVNSMTKELSDAEIKVFRP	S5926*
		EITVLRYPEERLYFPSAHKGCIAKIVDSIYY	S4048*

		QTYNSKMADQVSMSPSNQIVHEIVERVLHML	S3005*
		QKMVDSVYSNILQMSSSVDSIQKNIISRSPI	S4237*
		KMVDSVYSNILQMSSSVDSIQKNIISRSPIM	S4238*
		DSVYSNILQMSSSVDSIQKNIISRSPIMIDQ	S4241*
		TTSVITHLKNFKTGVSTEKFSHVSSLSKKID	S3483*
		NFKTGVSTEKFSHVSSLSKKIDDANQPLISI	S3492*
		EIPKEMCPQNLKNIASDILNMVFAKLEHFAS	S1578*
		KAGVKKPRRPRVRNLSDPEEKYRVTGTRPFN	S960*
		NFHKQSILSNKQDKNSFCQLLAKPCLQHSLA	S1404*
		KNSFCQLLAKPCLQHSLADEKDFKVSSKLEV	S1417*
		KVHYDISKSRFRRQASTMYTTMLSHVHLEKI	S4792*
		SRFRRQASTMYTTMLSHVHLEKIVTQLMSQI	S4800*
		RQGQSVPSQVNYQKTSLDYQFPKIGSSKSDS	S627*
		KTPLDEVSYMVKQVLSDVVESHKSQKPSPFG	S4298*
		KKVNDSTEESLKQVLSFIETGKRENPRVFHY	S3308*
		LAQKNKLNYLARRLNSLVGSLRSRESKEVVN	S3699*
		CLKRTGCSIAELRRNSIRGIGPSSTDVSEMG	S6664*
		SNTSDTPRTRRSSQGSVSGMGFSSEDDMKEK	S3203*
		TRRSSQGSVSGMGFSSEDDMKEKYSVTSNSS	S3211*
		KKHTQFGKKHAGKSISLSFSDGKESRGINTA	S3952*
		HTQFGKKHAGKSISLSFSDGKESRGINTACF	S3954*
		QFGKKHAGKSISLSFSDGKESRGINTACFHK	S3956*
		ELDMPSCKSLAEETTSRDKLQYKEEEILASE	S6859*
		GNKSSLIEKSLHSQLSTYTMKIVNIILHAIQ	S1289*
		SSQEPLDFSRPIYSRSLTSDKALVILKDSAQ	S5803*
		EPLDFSRPIYSRSLTSDKALVILKDSAQLVP	S5806*
		TSPPTSPPATMKSQGSQVQQLATSPPTSMKS	S5528*
		ATMKSQGSQVQQLATSPPTSMKSQRIQVQQS	S5536*
		QQFATSPPTSMKSQGSQVQQSAMSPPTTMKS	S5582*
		TSMKSQGSQVQQSAMSPPTTMKSRGAQVQES	S5590*
		TSTYTDKTLSTSICESGNFHKQSILSNKQDK	S1387*
		VAKTVENSPEKEVFISDLKIQNEKKLDVSKS	S5706*
		KTVNSVYGNIFYEYKSLTQLHRAVQYDTSGF	S5049*
		EKEDEVQAAKEPAPTSPPEVKETVTKEPSPS	S5957*
		KFLEDVTSGLLAKVFSKLSNVKTKLTPENVL	S6123*
		DGVSSVPSCRVITESSYTAAHGDLDTNRIIE	S6568*
		FREIPEECQHVKVLPSVQGGGFDYREAPMDC	S3812*
		IYPDKLVGETVTRVLSKIFSPNTNINVELEN	S4329*
		SKDVEQKVQNVVQTVSKECQTASPYTVQLPY	S6092*
		VQNVVQTVSKECQTASPYTVQLPYKFLEDVT	S6099*
		CLQHSLADEKDFKVSSKLEVLDQIGDTLHEM	S1428*
		KIKVVQPVKESEAVPSADKAAIEKVVNSVYT	S6337*

		PFKVWFDSEKKMKYLSSLNMEHEKTSQLRSG	S1226*
		GFSSEDDMKEKYSVTSNSSSHISSCVENTNK	S3223*
		SSEDDMKEKYSVTSNSSSHISSCVENTNKSL	S3225*
		SNLKRFLALSKCCQPTSGENIESIEESGIQI	T6815*
		AKKEDESKVKPATRETTSPPTSPPATMKSQG	T5512*
		SCSHQNTETIDKDPPTMSNTQLISKAILENV	T1472*
		TSVITHLKNFKTGVSTEKFSHVSSLSKKIDD	T3484*
		VHYDISKSRFRRQASTMYTTMLSHVHLEKIV	T4793*
		KLSAGEKTPRESRSKTALGLPQTPQVEDTKT	T2721*
		DAENISQRVQLLPKETSMQISGALKQNQLAL	T5441*
		QTYQRQGQSVPSQVNYQKTSLDYQFPKIGSS	Y623*
P34152	Focal adhesion kinase 1	SEPTTWASIIRHGDATDVRGIIQKIVDSHKV	T62*
Q61345	Forkhead box protein D1	AGTGGGAKNPLVKPPYSYIALITMAILQSPK	Y133*
A2APV2	Formin-like protein 2	LICDQERFQVKNPPHTYIQKLKGYLDPAVTR	T78*
		ICDQERFQVKNPPHTYIQKLKGYLDPAVTRK	Y79*
		QVKNPPHTYIQKLKGYLDPAVTRKKFRRRVQ	Y86*
P05064	Fructose-bisphosphate aldolase A	CQGKYTPSGQSGAAASESLFISNHAY_____	S354*
Q8R2K1	Fucose mutarotase	WKRYESLLLEADCKKTLMKLERFEFYERAKK	T110*
O70291	G protein-coupled receptor kinase 4	FRRLFRRTGCLNIALSEEREPTEH_______	S566*
P26048	Gamma-aminobutyric acid receptor subunit alpha-2	SVMIQNNAYAVAVANYAPNLSKDPVLSTISK	Y374*
Q6PGG2	GEM-interacting protein	LRRNPLPKHFEITQETARLLSKLNSDAVSRT	T941*
O09172	Glutamate--cysteine ligase regulatory subunit	MGTDSRAAGALLARASTLHLQTGNLLNWGRL	S16*
		TGNLLNWGRLRKKCPSTHSEELRDCIQKTLN	S37*
P48774	Glutathione S-transferase Mu 5	QIMDFRMQLVRLCYNSNHENLKPQYLEQLPA	S121*
		KPQYLEQLPAQLKQFSLFLGKFTWFAGEKLT	S142*
		EEKRYICGEAPDYDRSQWLDVKFKLDLDFPN	S47*
		___________MSSKSMVLGYWDIRGLAHAI	S5*
		______________MSSKSMVLGYWDIRGLA	S2*
		_____________MSSKSMVLGYWDIRGLAH	S3*
		PNLPYLMDGKNKITQSNAILRYIARKHNMCG	S76*
Q9D4P7	Glutathione S-transferase theta-4	PFDFQFVDLLKGHHHSKEYIEINPLRKLPSL	S42*
Q64467	Glyceraldehyde-3-phosphate dehydrogenase, testis-specific	______________MSRRDVVLTNVTVVQLR	S2*
		GKLTGMAFRVPTPNVSVVDLTCRLAKPASYS;GKLTGMAFRV PTPNVSVVDLTCRLEKPAKYD	S239*
		PELKGKLTGMAFRVPTPNVSVVDLTCRLAKP;PELNGKLTGMA FRVPTPNVSVVDLTCRLEKP	T235*
Q8CI94	Glycogen phosphorylase, brain form	GEGFLTDLSQLKKLLSLVDDEAFIRDVAKVK	S524*
P16627	Heat shock 70 kDa protein 1-like	NEPTAAAIAYGLDKGSHGERHVLIFDLGGGT	S191*
P48722	Heat shock 70 kDa protein 4L	AQNKLSLTQDPVVKVSEIVTKSKELDNFCNP	S769*
Q9DAM3	Heat shock protein beta-9	SFSTGQREPGENRVASRCPSVALAERNQVAT	S22*
		GQREPGENRVASRCPSVALAERNQVATLPVR	S26*
		PPTLDPAAMTCSLTPSGHLWLRGQNKCLPPP	S126*
P17710	Hexokinase-1	EQHRQIEETLSHFRLSKQALMEVKKKLRSEM	S536*

		DGSEKGDFIALDLGGSSFRILRVQVNHEKSQ	S144*
		NKGTPRLRTTVGVDGSLYKMHPQYSRRFHKT	S471*
P70349	Histidine triad nucleotide-binding protein 1	PQAPTHFLVIPKKHISQISVADDDDESLLGH	S61*
Q9CQ70	Histone H2A-Bbd type 1	QNNEQLHQLFKQGGTSVFEPPEPDDN_____	S101*
		MARKRQRRRRRKVTRSQRAELQFPVSRVDRF	S16*
		VQNNEQLHQLFKQGGTSVFEPPEPDDN____	T100*
P62806	Histone H4	AKRHRKVLRDNIQGITKPAIRRLARRGGVKR	T31*
O88491	Histone-lysine N-methyltransferase, H3 lysine-36 and H4 lysine-20 specific	VGELIDEEECRARIRYAQEHDITNFYMLTLD	Y1885*
Q80W93	Hydrocephalus-inducing protein	AERERLEKLKALEERSDVEGEGEEEHEGKKD	S2585*
Q9QZ29	Immunoglobulin-binding protein 1b	_____________MASFTEEMQKPKLRELLE	S3*
		EVSPTLQEAEKQAPPSETFTVSEKEEPDLEQ	S295*
Q6NVF0	Inositol polyphosphate 5-phosphatase OCRL-1	CDRILWRGINVNQLHYRSHMELKTSDHKPVS	Y512*
Q8CIM8	Integrator complex subunit 4	EEIATKKLRLTKPSKSAALHIDLCKATSPAD	S37*
Q8C262	Interferon-inducible GTPase 5	PAHRRHAGLLSLPDISLEALQKKKDMLQEQV	S251*
		TRLEVGVTGESGAGKSSLINALRGLGAEDPG	S66*
		RLEVGVTGESGAGKSSLINALRGLGAEDPGA	S67*
		RSLRGYHRSFGLDDDSLAKLAEQVGKQAGDL	S310*
		ALRTAFESGDIPQAASRLRELLANSETTRLE	S39*
P97378	Interleukin-12 receptor subunit beta-2	AKGKYDLRDLRPFTEYEFQISSKLHLSGGSW	Y306*
Q6NWV3	Intraflagellar transport protein 122 homolog	RSKPFHDSEELVPLCYRCSTNNPLLNNLGNV	Y999*
Q9D0P8	Intraflagellar transport protein 27 homolog	EQSFISCTKWLEKVRSQTSGISLPGVLVGTK	S109*
		FISCTKWLEKVRSQTSGISLPGVLVGTKTDL	S112*
A6H690	IQ and AAA domain-containing protein 1-like	QQMLADILNRLEPLVSQENLRGLSRTAALIL	S151*
		MPMPSLFDIRQNMALYGVLRLGSHDIHTMAP	Y548*
Q8CDK3	IQ and ubiquitin-like domain-containing protein	NDDEVEDIRSKHSTKSPPKIIITRRIQPH__	S775*
		______________MSDPEEERVADSTAHYE	S2 *
Q80W32	IQ domain-containing protein G	IPVQYERRQSLTQKASHEGASMITTTPKKST	S93*
		ASHEGASMITTTPKKSTSLLTKEKSMMAENK	S107*
		SILGYIIPVQYERRQSLTQKASHEGASMITT	S87*
Q9D5V2	Kelch-like protein 10	AASTRFHQPHMERKMSAMTCEIFNELRLEGK	S22*
P59280	Kelch-like protein 8	KLISCHKLVLACVIPYFRAMFLSEMSEAKQA	Y100*
Q9WV04	Kinesin-like protein KIF9	ARERETSSIEPLISDSPKEELRAPRPSTPPS	S561*
P29812	L-dopachrome tautomerase	LGLLAFLQYRRLRKGYAPLMETGLSSKRYTE	Y500*
P06151	L-lactate dehydrogenase A chain	KLVIITAGARQQEGESRLNLVQRNVNIFKFI	S105*
		VKEQLIQNLVPEDKLSRCKITVVGVGNVGMA	S19*
		______________MSTVKEQLIQNLVPEDK	S2*
Q9D4C1	Lamin tail domain-containing protein 1	SSTNEEKEEPIQKTPSQVYPVLYREKEIPPT	S339*
		KVQDGEIRKEKIGTITPSKQHSSVHFFPKIM	T36*
Q0VET5	Lamin tail domain-containing protein 2	SGEVLTQQEGVKAETSELLPVIPECPSRLCL	S593*
		QVLSEHQATPCVTLGSKIFTDNTDWSIDCFP	S487*
Q9JJK2	LanC-like protein 2	ERSFPNPFPDYEAAASAAGLAAGSAEETGRV	S35*

Q9DAE3	Late cornified envelope-like proline-rich protein 1	CPQKGCVKPCPPKCPSPCLPRK__________	S114*
		TPTSPLCPPLCSPRCSGTLACCPPSCPQKGC	S89*
Q69ZB0	Leucine-rich repeat and coiled-coil domain-containing protein 1	NRKMRQPYLRDLYVRSSLVNCNNLRDLDEQK	S386*
		RKMRQPYLRDLYVRSSLVNCNNLRDLDEQKT	S387*
		HIWNLRHLDLSSNQISQIEGLNTLTKLCTLN	S73*
		IHAINLHCNNISKISSIDHIWNLRHLDLSSN	S55*
		RKVWGQELACQSSTLSQSRGKLEAQIESLCR	S775*
		QIKLLQEQIALIEKCSQEQLNEKSSQLDSIV	S848*
		KCSQEQLNEKSSQLDSIVEKLERHNERKEKL	S861*
		LDEQKTGVIKVDKNFSDNSTYRSLVEQLDQE	S412*
		QKTGVIKVDKNFSDNSTYRSLVEQLDQEREM	S415*
Q9DAK8	Leucine-rich repeat-containing protein 51	RLGEVNKLAVLPRLRSLTLHGNPIEEEKGYR	S131*
C8YR32	Lipoxygenase homology domain-containing protein 1	FPCDRWLATSEDDKKTIRELVPYDIFTEKYM	T1221*
		DDKKTIRELVPYDIFTEKYMKDGSLRQVYKE	T1232*
Q9D4H7	LON peptidase N-terminal domain and RING finger protein 3	IATFLPEEFKERKRLYEEEMEELSNLNKNVP	Y536*
Q9DB27	Malignant T-cell-amplified sequence 1;Malignant T-cell-amplified sequence 2	LPHQQVDKGAIKFVLSGANIMCPGLTSPGAK	S107*
Q69ZF7	Metal transporter CNNM4	VVITKFLRRSENMKLYALCTRPRADGPWTRW	Y145*
Q91VN4	MICOS complex subunit Mic25	MDEEERVRVLQGIRLSESVVNRMKDCSQPSA	S31*
Q0P557	Mitochondria-eating protein	RGAFWSSVRPVMRCRSRSLSPICPRNHFGIS	S504*
		YEKHLQNLKEEIAVLSAEKSGLQGRSARSPS	S231*
		LQNLKEEIAVLSAEKSGLQGRSARSPSPSTG	S235*
		TIKCRLLPLLQTSFSSVNMGKTAESEMCATQ	S88*
		SRSLSPICPRNHFGISTVSRSRSPSPIRCTF	S519*
		RSTKRTQDQRPQDVVSNYEKHLQNLKEEIAV	S214*
		LQTSFSSVNMGKTAESEMCATQDFQLRSKNR	S97*
		EGVETIKCRLLPLLQTSFSSVNMGKTAESEM	T84*
		LAESGKSLEGAKNGSTISLLAAEEEINQLKK	T155*
		RSLSPICPRNHFGISTVSRSRSPSPIRCTFA	T520*
Q9WV98	Mitochondrial import inner membrane translocase subunit Tim9	KDFTTREVKPEEVTCSEHCLQKYLKMTQRIS	S49*
Q9CYG7	Mitochondrial import receptor subunit TOM34	YRRAQAYKALKDYKSSLSDISSLLQIEPRNG	S280*
Q61532	Mitogen-activated protein kinase 6	QFHSPVGSPLKSIQATLTPSAMKSSPQIPHK	T695*
O70451	Monocarboxylate transporter 2	KSASHASREMEALNRSKQDEVTVKASNAHNP	S459*
		LLDKERKREKAKKKKSASHASREMEALNRSK	S445*
		DKERKREKAKKKKSASHASREMEALNRSKQD	S447*
		RKREKAKKKKSASHASREMEALNRSKQDEVT	S450*
B2RPV6	Multimerin-1	HLEGALRQEHSQIVLYHQSLNETLSKMQEAH	Y486*
Q5SUV2	MYCBP-associated protein	KETVYLDLPDEQGQKSPPVTESKVTSGKAGK	S797*
		PSLRTPVPLLLVEKASGSISPRNLVSEYSQL	S628*
Q9QWV4	Myeloid leukemia factor 1	RGRTHNRRERDDGEDSLTHADVNPFQTMDRM	S63*
		__________MFRMLSSSFEDDPFFADSFLA	S6*

		RMLSSSFEDDPFFADSFLAHRESMRNMMRSF	S18*
		EDDPFFADSFLAHRESMRNMMRSFSEPLGRD	S25*
		REKIHRNSAIESGRRSNVFVDKLNVKGSPVK	S248*
		LKYKSIGRSGNTGMRSVGHEHPGSRELKRRE	S219*
		SGNTGMRSVGHEHPGSRELKRREKIHRNSAI	S227*
		GRRSNVFVDKLNVKGSPVKITKK________	S260*
		RTHNRRERDDGEDSLTHADVNPFQTMDRMMA	T65*
Q62234	Myomesin-1	GKATGHSTLVLIGDVYKKLQKEAEFQRQEWI	Y1320*
O08638	Myosin-11	LEEQVEQEAREKQAATKSLKQKDKKLKEVLL	T1841*
Q9JIL4	Na(+)/H(+) exchange regulatory cofactor NHE-RF3	EEHAQVVELVRKSGNSVTLLVLDGDSYEKAV	S81*
		NASHEEVVEKVTKSGSRIMFLLVDKETARCH	S205*
Q3TKR3	NACHT, LRR and PYD domains-containing protein 4C	TLHVLGLRITDFDKETQELLMAEEEKNPHLS	T962*
Q8BHG1	Nardilysin	LMIRDAIDREVEAVDSEYQLARPSDANRKEM	S324*
P97798	Neogenin	ADNSLPKHQKITDSRYYTVRWKTNIPANTKY	Y921*
Q6ZWR6	Nesprin-1	ASKSFVHRVSQLSSQYLALSNVTKEKVSRLD	Y3259*
Q8CAF4	NHS-like protein 1	GSDEVLTPTKPRTTEDLFAAIHRSKRKVLGR	Y1346*
Q9D6Z1	Nucleolar protein 56	SSAKRKKSSPKEEVASEPEEAASPTTPKKKR	S536*
Q6ZQH8	Nucleoporin NUP188 homolog	SFYNELHKHKPHDVLSHEDGTLWRRQTPKLL	S487*
Q63912	Oligodendrocyte-myelin glycoprotein	DHKQNITYLLKWVMETKAHVIGTPCSKQVSS	T245*
		YLLKWVMETKAHVIGTPCSKQVSSLKEQSMY	T252*
Q9R109	Ornithine decarboxylase antizyme 3	DPVQLDFHFRLTPHSSAHWHGLLCDHRLFLD	S143*
		__________MPCNRSRPSLYSLSYIKRGKT	S6*
		__MPCNRSRPSLYSLSYIKRGKTRNYLYPFW	S14*
		_____MPCNRSRPSLYSLSYIKRGKTRNYLY	Y11*
Q8R554	OTU domain-containing protein 7A	SPADKGSGSRGDAWKYSTDVKLSLNILRAAM	Y617*
Q61999	Outer dense fiber protein 1	NRILASSCCSSNILGSVNVCGFEPDQVKVRV	S114*
		CDLHPYPYCLCYSKRSRSCGLCDLYYPCCLC	S62*
		VCVSAERENRYDCLGSKKYSYMNICKEFSLP	S149*
A3KGV1	Outer dense fiber protein 2	RGHLQAQLRCKEAENSRLCMQIKNLERSGNQ	S361*
		LQGYERKNIDLTAIISDLRSRIEHQGDKLEM	S632*
		AEYSAFKLENERLKASFAPMEDKLNQAHLEV	S514*
		DKLNQAHLEVQQLKASVKNYEGMIDNYKSQV	S535*
		AENSRLCMQIKNLERSGNQHKAEVEAIMEQL	S373*
		KASVKNYEGMIDNYKSQVMKTRLEADEVAAQ	S548*
		RDSLVERLHRQTAEYSAFKLENERLKASFAP	S502*
		___________MSASSSGGSPRFPSCGKNGV	S5*
		_______MSASSSGGSPRFPSCGKNGVTSLT	S9*
		KAQAKTASELSKSMESMRGHLQAQLRCKEAE	S344*
		VLRTPCGAPSVTVTKSHKRGMKGDTVNVRRS	S43*
		ERKEELEEVAHELAETEHENTVLRHNIERIK	T172*
		LEEVAHELAETEHENTVLRHNIERIKEEKDF	T177*
		GKNGVTSLTQKKVLRTPCGAPSVTVTKSHKR	T31*

		ALKDTIGKLKTEKQMTCTDINTLTRQKELLL	T242*
Q920N1	Outer dense fiber protein 3	VSQPSFSIKGRSKLGSFSDDLHKTPGPAAYR	S171*
		KGRSKLGSFSDDLHKTPGPAAYRQTEVQVTK	T179*
Q5M8M2	Outer dense fiber protein 3B	______________MGSEVWVGTWRPHRPRGP	S3*
		VISKVSAPTYSIYSRSPVGSCFEDLSKTPGP	S169*
		SRVISKVSAPTYSIYSRSPVGSCFEDLSKTP	S167*
Q8VI88	Outer dense fiber protein 4	IRTSRNRRSLEHRRNSLLPFQWKATNNSRWM	S28*
Q8JZM7	Parafibromin	______MADVLSVLRQYNIQKKEIVVKGDEVI	Y11*
Q9DAK2	Parkin coregulated gene protein homolog	TKLLPQQTFQVHQPRSLVSEGFTVKAMMKNS	S20 *
		LPQQTFQVHQPRSLVSEGFTVKAMMKNSVVR	S23 *
Q9QYC1	Pecanex-like protein 1	YVTTSSKLEEWLANETMQEGLRLCADRNYVD	T1725*
A2AR02	Peptidyl-prolyl cis-trans isomerase G	KGDKSELNEIKENQRSPVRVKEKKITDHRHM	S395*
Q62009	Periostin	ISINGIKMVNKKDIVTKNGVIHLIDEVLIPD	T355*
P70296	Phosphatidylethanolamine-binding protein 1;Hippocampal cholinergic neurostimulating peptide	WHHFLVVNMKGNDISSGTVLSDYVGSGPPSG	S99*
Q66GT5	Phosphatidylglycerophosphatase and protein-tyrosine phosphatase 1	NWSPEEAIEAIAKIRSHISIRPSQLEVLKEF	S167*
		PEEAIEAIAKIRSHISIRPSQLEVLKEFHKE	S170*
		IEAIAKIRSHISIRPSQLEVLKEFHKEITAR	S174*
P09041	Phosphoglycerate kinase 2	DVYVNDAFGTAHRAHSSTVGVNLPQKASGFL	S174*
		VYVNDAFGTAHRAHSSTVGVNLPQKASGFLM	S175*
		RAHSSTVGVNLPQKASGFLMKKELDYFSKAL	S186*
		AKGTKALMDEVVKATSNGCVTIIGGGDTATC	S364*
		VEEEGKGKDSSGKKISADPAKVEAFQASLSK	S141*
Q8CHP8	Phosphoglycolate phosphatase	STCSLKTILTLTGVSSLEDVKSNQESDCMFK	S285*
Q6UQ17	Phospholipid-transporting ATPase IK	ISILAKKRRHFFGKGSQEEVHPNTSSQTMEK	S1240*
		SQMWRNAGPSLGTSHSADSKIRESPEVQRER	S800*
		WRNAGPSLGTSHSADSKIRESPEVQRERAFV	S803*
		FRNGMLNVSPIGRSCSQEERIEFYELDKKEH	S133*
Q99K85	Phosphoserine aminotransferase	KSQMIYEIIDNSQGFYVCPVERQNRSRMNIP	Y289*
Q99LS3	Phosphoserine phosphatase	PFKDALTQRLALIQPSRDQVQRLLAEHPPHL	S72*
Q61233	Plastin-2	KIKVPVDWNRVNKPPYPKLGGNMKKLENCNY	Y447*
Q8BHD1	POC1 centriolar protein homolog B	STTVKKRPEDVSDVPSESLRSVPLAVADALE	S416*
Q9WVQ0	Polyamine-modulated factor 1-binding protein 1	ERSLNLYRDKYQTSLSNIELLECQVKMLEGE	S204*
		CTTKELDTSLQEARQSTSKIDCEALRAEIQK	S465*
		TKELDTSLQEARQSTSKIDCEALRAEIQKLK	S467*
		SPCVIQEHQETLKRLSEVWQKVSEQDDLIQE	S257*
P22907	Porphobilinogen deaminase	RVGTRKSQLARIQTDTVVAMLKALYPGIQFE	T37*
Q9D529	Postacrosomal sheath WW domain-binding protein	RWAAIPHGESLLKKCSEVDLSFPQSPPGSNL	S27*
Q99PV0	Pre-mRNA-processing-splicing factor 8	EGECNVMLESRFEKMYEKIDLTLLNRLLRLI	Y985*
O35286	Pre-mRNA-splicing factor ATP-dependent RNA helicase DHX15	RFNLPRRSTDFTSRDYYINIRKALVTGYFMQ	Y685*
Q9D0R4		LKEIKEELLHSEKLKTYFEDNPRDLQLLRHD	T462*

	Probable ATP-dependent RNA helicase DDX56	KEIKEELLHSEKLKTYFEDNPRDLQLLRHDL	Y463*
P0CW70	Probable C-mannosyltransferase DPY19L2	KLPSGARNYAARRIQSLNAQNYFQLEEVAKL	S76*
P70398	Probable ubiquitin carboxyl-terminal hydrolase FAF-X	QQNEQSESEKAGSTKYRLVGVLVHSGQASGG	Y1863*
P50580	Proliferation-associated protein 2G4	______________MSGEDEQQEQTIAEDLV	S2*
Q9D9B7	Proline-rich protein 30	SPHSCQSPSRLQDLQSPKITSPVPSSPSPRI	S154*
		LHLLPPKNSTPVGVESQEEALQRQKSTIQES	S323*
		RGQARALHLLPPKNSTPVGVESQEEALQRQK	T317*
P99026	Proteasome subunit beta type-4	VERCMRVLYYRDARSYNRFQIATVTEKGVEI	Y229*
O70552	Protein BTG4	IETFALKLMTILFEKYRGHWHPDCPSKGQAF	Y42*
Q9DAF3	Protein DDI1 homolog 1	TPGRTPNHPRADFTGSGSAVPGTSSSRHPHQ	S99*
		HHQRIPSTQQAHGLASGENMTFAQELDSPAL	S136*
		SIDLKKNVLVIGTTGSQTHFLPEGELPLCAK	S362*
Q99LT0	Protein dpy-30 homolog	TQVAENPHSEYGLTDSVERIVENEKINAEKS	S26*
Q8CJF7	Protein ELYS	DIMYSFPNKTDTPIESFPTAFAISWGQVKLV	S797*
		KTDTPIESFPTAFAISWGQVKLVQGFWLLDH	S805*
		YSFPNKTDTPIESFPTAFAISWGQVKLVQGF	T800*
Q8BMB0	Protein EMSY	QSTKQQKLSQPQLEQTQLQVKTLQCFQTKQK	T912*
		KLSQPQLEQTQLQVKTLQCFQTKQKQTIHLQ	T918*
		LEQTQLQVKTLQCFQTKQKQTIHLQADQLQH	T924*
Q9D4K5	Protein FAM166A	LLTDPSVQKSPCSVLSPMTKPKFIEDFSKSK	S57*
Q66LM6	Protein FAM170A	KHLEIEESKEAGISKSQEDISHPESTGVPKA	S23*
		_MKRRQKRKHLEIEESKEAGISKSQEDISHP	S15*
		PIGEEKEHEEKPESDSPPGSPAVEERPRAKT	S198*
A2APU8	Protein FAM205A	EATTLEIRQLLQSKKSQISPALLGLPQRAAC	S123*
		TLEIRQLLQSKKSQISPALLGLPQRAACLEM	S126*
Q80YD3	Protein FAM205CP homolog	VENKKTSSSLLRHSRSFSCLEALSPPKVLND	S136*
Q8C627	Protein FAM221B	EKAIAEIPLEEFGYDSPISKVKEEPLQLSPT	S121*
		QVPESEHFPKHSFSESPTQAKEDESTKEGEA	S239*
		DTKGAEGLQDSSVSGTPPVKSSSSGLLSLPP	T80*
Q497Q6	Protein FAM228B	PKLKSSKEWLEPQSLSFMEALAKEDTDAAVQ	S33*
		________MTTMKNRSQDDMVTGTLPKLKSS	S8*
		DDEKRTLLQCETGKIYTMKEFKEIEKAQLHS	Y183*
Q5STT6	Protein FAM71B	VAAPSAGMTKAETATSPTSGVISLAATTTKP	S370*
		TLELTRLLPLKFVKISVHDHEKQQLRLKLAT	S138*
		HARRARFIKGKGCKPSKTLELTRLLPLKFVK	S121*
		TVISETMEKIILETKSI______________	S664*
		EKLVYLLRPPMTNCISNSTLPTGETSVDTKS	S191*
		_______MPGMKRTMSSECLLPYYTAHSYRS	S9*
		______MPGMKRTMSSECLLPYYTAHSYRSM	S10*
		GSVSKRATKITSFFRSFLVRPTPKAGDTSCD	S602*
		EKKQDIEAVMEKSKDSEFKDTVISETMEKII	S644*

		VMEKSKDSEFKDTVISETMEKIILETKSI__	S652*
		YYTAHSYRSMGVFNTSMGNLQRQLYKGGEYD	S31*
		DTGLNVAFAGSIKTKSPAEDKPEAPLVSTLQ	S464*
Q3TJZ6	Protein FAM98A	VYQPKRSLLSPKGKVSVAHLLAARQDLSKIL	S264*
Q8CB93	Protein GAPT	DVTKTVSSSAYVISPSMKASVESKGHKSTAK	S71*
Q5QNV8	Protein HEATR9	KAVKTEHTRFRKKPESPDDLCITDFRRAKLN	S494*
P16054	Protein kinase C epsilon type	TMTEKRILALARKHPYLTQLYCCFQTKDRLF	Y468*
P16054	Protein kinase C epsilon type	RILALARKHPYLTQLYCCFQTKDRLFFVMEY	Y473*
Q3U1D0	Protein Lines homolog	FLISSETCFLEYFVKYLKLLQKDWAHFLSIC	Y543*
Q8BRC6	Protein MAATS1	______________MSQTVTIQEPRPNDQRI	S2*
		LNVVLSPVKTQPLLLTPESSKYAIIPTKSTV	T173*
Q9D6Y4	Protein MEF2BNB	QLKGKKVTDKFTESVYVLANEPSVALYRLQE	Y22*
Q2YDW2	Protein misato homolog 1	LNPGTPLPSALHACASGEEVLAQYLQQQHPR	S431*
Q8BYB9	Protein O-glucosyltransferase 1	LILLSRKNPKLVDAEYTKNQAWKSMKDTLGK	Y236*
Q3KNI6	Protein PIH1D3	VNFPQPVERNSARASYIWEAETLEVRMTVQR	Y196*
Q8BSQ9	Protein polybromo-1	IRIRDELCKNGEILLSPALSYTTKHLHNDVE	S888*
B0QZF7	Protein PROCA1	ITIWRSESPIEKCQESNVIKDIKRKEKEQDE	S175*
		CERGKERPSSEDVVESLSPRKKEKTSSGQAK	S266*
		RGKERPSSEDVVESLSPRKKEKTSSGQAKKN	S268*
		QSVRGPVRTPESSPESPGGLESEYSCERGKE	S241*
		TRLPSWKRGYPASVDSSSDLFSFSEGENKET	S45*
		KRGYPASVDSSSDLFSFSEGENKETDRRCWK	S51*
		NHLSPSARPPDPNTGSATEVPDLSVPITIWR	S149*
		AKGKLTKKKTPVKSESSPADLSQSVRGPVRT	S219*
		RKIERTERTDITRLPSWKRGYPASVDSSSDL	S34*
Q8C8N2	Protein SCAI	DKSKQLFNGLRDLPQYGQKQWQSYFGRTFDV	Y82*
Q7TSD4	Protein TBATA	KPEYIGKAQVLLVHPSEDPEEKTTKAES___	S381*
		QTSQPTPSAYRFGRLSHHSFFSRHHPQPQRV	S78*
		QPTPSAYRFGRLSHHSFFSRHHPQPQRVTHI	S81*
		QGAKYSAETGRLIPASSQALTRRNRQGQRVH	S241*
		LQELQEPQETQEAAYSPSLKKTRSPPLPKTD	S350*
		______________MTTEVNQLSEHPLVSPK	T2 *
Q8R0E5	Putative uncharacterized protein ZNRD1-AS1 homolog	DTGDETKTKVQEEPPSPSPPPPPPPRRERQK	S135*
Q8VIG3	Radial spoke head 1 homolog	KNKKHGQGTFIYPDGSRYEGEWADDQRHGQG	S88*
Q9DA80	Radial spoke head protein 3 homolog B;Radial spoke head protein 3 homolog A	RALARKRAQEQLKPRTPEPVEGRKHVDIQTE	T100*; T227*
Q8CDR2	Radial spoke head protein 6 homolog A	FSQDTQHGPYLRDDPSLHLRPSDLGFMPIVG	S165*
		HGPYLRDDPSLHLRPSDLGFMPIVGEVPDPE	S171*
		KILQFLPERIFFRWHYQSIGSTLKKLLHTGN	Y106*
		LPSCEISVTRKVVQVYRKWILQNKPVFMEEP	Y430*
		QHETPLPRSWSPKDKYNYIGLSQGNLRVHYK	Y63*
		ETPLPRSWSPKDKYNYIGLSQGNLRVHYKGH	Y65*

		KYNYIGLSQGNLRVHYKGHGKNHKDAASVRA	Y77*
Q9CT10	Ran-binding protein 3	QKRSAGSSSPEAGEDSDHEDGNYCPPVKRER	S40*
Q91ZR1	Ras-related protein Rab-4B	SCLLHQFIENKFKQDSNHTIGVEFGSRVVNV	S37*
Q9ERD6	Ras-specific guanine nucleotide-releasing factor RalGPS2	SWTKYWAALCGTQLFYYAAKSLKATERKHFK	Y502*
		WTKYWAALCGTQLFYYAAKSLKATERKHFKS	Y503*
P31266	Recombining binding protein suppressor of hairless	QLHKCAFYLKDTERMYLCLSQERIIQFQATP	Y323*
G3UYX5	Regulator of G-protein signaling 22	CIPHHQTASPVIATASEIFDDGVHPRTIKSL	S241*
		SKKAEDTTGYAMAKLSLSDVSKQTEYWLNVP	S823*
		GSKRLPESTTVVRLTSFTDISECLKPQLERK	S617*
		GTQQLGRSEPLNAVSSKDGGLEKGSKRLPES	S594*
		YKVSHVIFDKVPPVPSNKAIVSPPVEMVEEI	S355*
Q8BRH3	Rho GTPase-activating protein 19	AFMIKHSQKLFKAPAYIRECARLYYLGSRTQ	Y313*
		KLFKAPAYIRECARLYYLGSRTQMSKDDLDL	Y321*
		KICKYPLQLAELLKYTHPQHRDFKNVEAALH	T235*
		QKICKYPLQLAELLKYTHPQHRDFKNVEAAL	Y234*
Q5HZJ0	Ribonuclease 3	RHAMANCFEALIGAVYLEGSLEEAKQLFGRL	Y1051*
Q8BUH7	Ring finger protein 26	CLCQACTEILMRHPVYHRNCPLCRRSILQTL	Y405*
Q7TT28	RNA exonuclease 1 homolog	KTTTTVTPKRIAHSPSLQSLKKPVIPKEFGG	S788*
Q6NZN0	RNA-binding protein 26	DVRKRKQEILEKHIETQKMLISKLEKNKTMK	T756*
Q9ESG2	Ropporin-1	FGAMSRGEIPPVRERSEQIPLSNWAELTPEL	S56*
		GEIPPVRERSEQIPLSNWAELTPELLKVLHS	S62*
Q8BIJ7	RUN and FYVE domain-containing protein 1	IAMKLLEKDTHEKQDTLVALRQQLEEVKAIN	T452*
Q8R127	Saccharopine dehydrogenase-like oxidoreductase	DLPKGGGVFTPGAAFSRTKLIDRLNKHGIEF	S406*
		KLRSVSCLKPVPIVGTKLKRRWPVSYCRELN	T227*
Q62217	Semaphorin-5A	___MKGACILAWLFSSLGVWRLARPETQDPA	S13*
P28661	Septin-4	FVKDFPGSEPYHSAESKTRVARPQILEPRPQ	S52*
		DDVEFRGSLWPQPSDSQQYFSAPAPLSPSSR	S89*
P50431	Serine hydroxymethyltransferase, cytosolic	_____MADRDATLWASHEKMLSQPLKDSDAE	S11*
		SDALTELGYKIVTGGSDNHLILMDLRSKGTD	S348*
Q8BG54	Serine palmitoyltransferase 3	VVAAQLIRSLKITMGYEGNIGGMERIQQLKE	S421*
Q8BMY7	Serine protease inhibitor Kazal-type 2	YSNECTLCMKIREDGSHINIIKDEPC_____	S76*
Q52KI8	Serine/arginine repetitive matrix protein 1	SAQEEPAAAPEPRKETESEAEDDNLDDLERH	T913*
Q924X7	Serine/threonine-protein kinase 33	TEFFASQERKKERNTSRESSLKDLSIRTSNV	S63*
Q8K1Y2	Serine/threonine-protein kinase D3	GTPAYLAPEVLRSKGYNRSLDMWSVGVIVYV	Y752*
Q9JLN9	Serine/threonine-protein kinase mTOR	REAIRVLGLLGALDPYKHKVNIGMIDQSRDA	Y897*
Q8CFH6	Serine/threonine-protein kinase SIK2	VESLQNKSYNHFAAIYFLLVERLKSHRSSFP	Y330*
P58389	Serine/threonine-protein phosphatase 2A activator	FAEHSNQLWNISAVPSWSKVNQGLIRMYKAE	S283*
		GISRSAAFVIAYIMETFGMKYRDAFAYVQER	T138*
		HGNAGISRSAAFVIAYIMETFGMKYRDAFAY	Y134*
Q8CDC0	Serpin B13	LGTRGATASELQKVLYTEQGTESSRIKSEEE	Y58*
Q3TD49	Signal peptide peptidase-like 2B	ATQGPVPPKAVGSSLSEQPPSEELAKSPLST	S523*

Q91Z67	SLIT-ROBO Rho GTPase-activating protein 2	TSPAKFKKDKEIIAEYDTQVKEIRAQLTEQM	Y17*
P62317	Small nuclear ribonucleoprotein Sm D2	EMWTEVPKSGKGKKKSKPVNKDRYISKMFLR	S87*
		SGKGKKKSKPVNKDRYISKMFLRGDSVIVVL	Y95*
Q8CFJ7	Solute carrier family 25 member 45	SSFRQEGIGVFFKGMTLNSARAFPVNAATFL	T263*
Q9CY18	Sorting nexin-7	SEMLVGVMKRRDQIQTELDSKVEALTYKKAD	T293*
Q62261	Spectrin beta chain, non-erythrocytic 1	PDEKSIITYVVTYYHYFSKMKALAVEGKRIG	Y276*
Q148B6	Speriolin	VSNIPEKIIQASLNPSNHKLDEDLCQTLTQR	S377*
Q9D9W0	Speriolin-like protein	LSPSELHSRIADRKLSPLLSPLQDSLADKTL	S136*
		LLEPREISRPKKVCFSESNLPTGDRSRRTYY	S166*
Q9DA48	Sperm acrosome membrane-associated protein 1	ELSSMRYKDSTSLDQSPTDIPVHEDDALSEW	S288*
		NWAAVKSFWGSKTSATEIQSELSSMRYKDST	T268*
Q8C9J3	Sperm flagellar protein 2	TEKKAGAPPAEPPAMSPPLSSEAEKDKELHQ	S847*
		WKELFFSGIPIYEQTSLTHGQTEPTEDHRAE	S420*
		MAKIQAAIIQIPKPESNRTLKAIEAQKLMKK	S165*
		TQKGKAQGGKVSVKKSPVGSAEVSPTPTAPP	S890*
		KAQGGKVSVKKSPVGSAEVSPTPTAPPPPKA	S894*
		GKVSVKKSPVGSAEVSPTPTAPPPPKAGTEE	S898*
C4P6S0	Sperm head and tail associated protein	YNEPRPPTSAPPQMKSPKSPESRRNPYKCRS	S457*
		PRPPTSAPPQMKSPKSPESRRNPYKCRSLDN	S460*
		SQRSCPPSPRLCYVTSPPLIHQAPRASPVTS	S183*
		PLLGRLYRQEPAGSSSPCFDRFSLQGSPSPH	S139*
		SPSPHQRNLCCNYIDSPESQRSCPPSPRLCY	S165*
		CNYIDSPESQRSCPPSPRLCYVTSPPLIHQA	S175*
		PRRIPTPPPPPPVLSSPPPPERCPFEPFSPL	S110*
		LRRGAFQSRSRSRSSSPLQSSTQDRNESTNM	S747*
		PLRPGLAISPPLAHRSVETRPLTPASISHRG	S250*
		TAVLKTVAPTSCPHSSPCNPALPSRYPKSSP	S542*
		SCLTISSPCLPRRIPTPPPPPPVLSSPPPPE	T100*; T227*
P15265	Sperm mitochondrial-associated cysteine-rich protein	SDSDTSGQTLEKGSQSPQSPPGAQGNWNQKK	S125*
		PQSPPGAQGNWNQKKSNK_____________	S141*
		______________MSDPSKTNQCPPPCCPP	S2*
		___________MSDPSKTNQCPPPCCPPKPC	S5*
Q62252	Sperm surface protein Sp17	AAAYFENLLEKREKTSFDPAEWGAKVEDRFY	S54*
		SSGREETPVTPFEESTEEEREQEEAAALKIQ	T105*
Q8K450	Sperm-associated antigen 16 protein	DMGHIQVPVIKGSYESASITRESGDRAGHSC	S275*
Q5S003	Sperm-associated antigen 17	QNFTEKFKKYTKKSASQNEIEDLIKSTKESV	S2069*
		KKELDTIVPPNLQSRSWERFPSVEKKTPGPP	S1737*
Q9JLI7	Sperm-associated antigen 6	NCYPEEIVRYYSPGYSDTLLQRVDSYQPL__	S494*
Q32MG2	Spermatid-associated protein	VKRDNTTLPAQRSKESTLQLIREENRALQQL	S242*
Q2MH31	Spermatid-specific manchette-related protein 1	CVDPLPGRLPYQGYESPCSGRHYCLRGMDYC	S197*
		PLPGRLPYQGYESPCSGRHYCLRGMDYCTTR	S200*

		TTREPSTERRLRPLCSQQPTECVALRSPARN	S228*
		MFLFSRKTKTPISTYSDSYRAPTSIKEVYKD	S16*
		REVVVNMLNSLSRNRTLPQIVPRCGCVDPLP	T172*
Q9DAQ9	Spermatogenesis-associated protein 19, mitochondrial	GHDIHVTRDLVKHHLSKSDMLTDPSQEVLEE	S82*
		LVKHHLSKSDMLTDPSQEVLEERTRIQFIRW	S91*
		NKTEEEASRSIREKMSINDSPTHGHDIHVTR	S59*
		EEASRSIREKMSINDSPTHGHDIHVTRDLVK	S63*
		VMDDVMQERIDQVRRSVSHLMCDSYNDPSFR	S133*
		DDVMQERIDQVRRSVSHLMCDSYNDPSFRTS	S135*
		RIDQVRRSVSHLMCDSYNDPSFRTSCSEC__	S141*
		IQFIRWSHTRIFQVPSEVMDDVMQERIDQVR	S116*
		QEVLEERTRIQFIRWSHTRIFQVPSEVMDDV	S107*
		VVQHWLNKTEEEASRSIREKMSINDSPTHGH	S53*
		TRDLVKHHLSKSDMLTDPSQEVLEERTRIQF	T88*
		ASRSIREKMSINDSPTHGHDIHVTRDLVKHH	T65*
Q80YT5	Spermatogenesis-associated protein 20	PSANSVSAHNLLRLHSFTGHKDWMDKCVCLL	S662*
Q9D9T6	Spermatogenesis-associated protein 3	ASSSRNCCCGSGGLGSCLLHG__________	S188*
E9QAF0	Spermatogenesis-associated protein 31	LNLVNSQNRTIVPGNSNHHLYCPRHCALQRD	S982*
Q8C5V0	Spermatogenesis-associated protein 32	QATQITQISSEKQDESPKSMAHKSWTRETRN	S251*
		ATIQGEVKFVQTPIASPQLQEAKEDSVPGTK	S297*
		QTSKHLFWSNKLIQASEHSLQKALEKHHRSP	S135*
		KHLFWSNKLIQASEHSLQKALEKHHRSPQEK	S138*
		MAHKSWTRETRNVACSYLDINQAGLKTATIQ	S270*
		EEEMGGRYRSIPVQTSKHLFWSNKLIQASEH	S122*
		EPELPSKTTPETEGDSYTESPEQQNYRMESL	S87*
		GLKTATIQGEVKFVQTPIASPQLQEAKEDSV	T293*
Q8K3V1	Spermatogenesis-associated protein 4	SDNDGNARKEIHVKQSGNPCENTENL_____	S285*
Q3U6K5	Spermatogenesis-associated protein 6	ASPVLNRASLRERFHSDWCSPPNCDEIHDRV	S368*
		ISSRKCRTQDKFTYHSAPVEKSHGRLQCRTS	S169*
		RTQDKFTYHSAPVEKSHGRLQCRTSRSQKKK	S175*
		QRPRGSFHLDDGECWSNRAASCKGKSHRPVF	S447*
		SFHLDDGECWSNRAASCKGKSHRPVFENSMD	S452*
		CINTKNYEQPTISSKSHSPSPYTKRRMCELS	S215*
		DERDPEREDELELKRSLLYRDSAYDSDPEYS	S414*
		CEEYLSPRTCSKPQHSARTLLVHSAPSTTPK	S335*
		KPPFVIRHVDPPSPRTDNFFGSPGRDCERDG	T268*
		YLSPRTCSKPQHSARTLLVHSAPSTTPKHCA	T338*
		PQHSARTLLVHSAPSTTPKHCASPVLNRASL	T347*
Q80VP2	Spermatogenesis-associated protein 7 homolog	LHFQKALISRENEFVSDEETVSHHERQQYQE	S442*
		VALAKTPSGDLLEKHSDLFSNKQSPFTPRTL	S216*
Q9D9R3		KPMFSESEIKAKPYRSLPEKPDNLLDQPKPP	S214*

	Spermatogenesis-associated protein 9	GTLIQGLNHISSSSKSVAKLLQPRLAYRLLE	S88*
Q9D5N8	Spermidine/spermine N(1)-acetyltransferase-like protein 1	FDLRQAGPIELGKKESDTWQLVPSQQGKTPS	S246*
Q8BZX4	Splicing regulatory glutamine/lysine-rich protein 1	PVKEKEHSKESDATVSKAADEKGSPRTEDEG	S457*
		TTPVKEKEHSKESDATVSKAADEKGSPRTED	T455*
Q9Z0G2	SRSF protein kinase 3	ATEWQQSGAQPPSRSTVSTAPQEVLIGKLSK	T248*
		WQQSGAQPPSRSTVSTAPQEVLIGKLSKNKR	T251*
F6XZJ7	Sterile alpha motif domain-containing protein 15	YEVTKPKFPDRKLRKSTEEADLKPHFKSTEQ	S168*
		SDSDESLSLQPKRTKSGKLHNANADTLFEVS	S25*
		STVLPQEVELLGRKETKPSLTPEFEHLTWSP	T467*
Q70E20	Sushi, nidogen and EGF-like domain-containing protein 1	AYVINVTTSQSTKSRYIPNGKLVSYTVRDLM	Y1150*
		QSTKSRYIPNGKLVSYTVRDLMPGRRYQLSV	Y1159*
Q64704	Syntaxin-3	APIPEPKTKDDLEQLTTEIKKRANNVRNKLK	T81*
		PIPEPKTKDDLEQLTTEIKKRANNVRNKLKS	T82*
O70439	Syntaxin-7	QDSPELRQLLQQKQQYTNQLAKETDKYIKEF	Y58*
P80318	T-complex protein 1 subunit gamma	DCKDPKACTILLRGASKEILSEVERNLQDAM	S380*
Q01755	T-complex protein 11	PSSHRLGMTERVHDASKLDCQLEERSLSSSS	S128*
Q5SVR0	TBC1 domain family member 9B	SGMYHGDLTEKLKALYKLHLPPALIPEEAES	Y925*
Q922G7	Tektin-2	VPQVDTFTRTTNRTLSPLKICQLELT_____	S420*
		ETLEIDRGCLSLNLTSPNISLKVNPTRIPKD	S196*
Q9JMB1	Testicular haploid expressed gene protein	LATPKVRNNIWSINMSEVSQVSRAAQMAVPT	S242*
Q9DA15	Testicular haploid expressed gene protein-like	GKDDLFPNAVIMTSPSLIARYLPRLQLASLR	S147*
Q8R0C3	Testis anion transporter 1	IPYTVSSTSQRNIVQSYEDTEKAWLPNSPPR	S673*
Q3URQ0	Testis-expressed sequence 10 protein	GRLGSSLAVTLIGILYMRSSFSGWSHSVNDC	Y657*
Q0VB26	Testis-expressed sequence 26 protein	SVSYKDRQILERFIHSHCDIEAKPNKREKQS	S276*
Q9DA77	Testis-expressed sequence 29 protein	PEPIREPPPPVKKTESPIEAGCCLWMKSKPA	S138*
Q9D9J2	Testis-expressed sequence 33 protein	RETFREGCVGEEGQDSRSPEQRTVPLSKKDS	S113*
		SQSPLGSSLGQGYLETPPLPPTPTCRTSLAM	T63*
Q9D9I1	Testis-expressed sequence 43 protein	_______________MASEKDDGPALPKLDDDN	S3*
		KDDGPALPKLDDDNQTAENTCKPAEEQPQQL	T20*
Q6NY15	Testis-specific gene 10 protein	NLEALLVANRDKEYQSQIALQEKESEIQLLK	S581*
		DQEIEMMENELDSARSEIELLRSQMTNERIS	S548*
		PERAHHRSPDRGLDRSLEENLCYRDF_____	S687*
Q61241	Testis-specific serine/threonine-protein kinase 1	SELNRDTEEGHPQQPSETHT____________	S361*
		KRCLRDDSGRLILSKTFCGSAAYAAPEVLQG	T174*
		RDTEEGHPQQPSETHT________________	T365*
O54863	Testis-specific serine/threonine-protein kinase 2	KRCLRDGSGRIVLSKTFCGSAAYAAPEVLQG	T174*
Q925K9	Testis-specific serine/threonine-protein kinase 6	________________MSGDKLLSELGYKLGRT	S2*
		FGFGRQAHGYPDLSTTYCGSAAYASPEVLLG	T170*
Q6IMH0	Testis, prostate and placenta-expressed protein	PSLRRMDMDTVKGCLSDEHCQSSTYFSKDDF	S62*
P70385	Testosterone 17-beta-dehydrogenase 3	VEYRDKGIIQVLTPYSISTPMTKYLNNKMT	Y225*

Q8C0S4	Tetratricopeptide repeat protein 21A	HHVLQAANVGLERYSSDPALQFFRAYGILGE	S37*
Q9D4B2	Tetratricopeptide repeat protein 25	VGIQKAQEAINNSVGSPSSIKLENKGDLSFL	S131*
		SSGGLSKKLLGDGHSSNLGIRRESREIYRRL	S572*
		DRKLMQEKWLRDRKRSPSQTAHYILKSLEDI	S264*
		KLMQEKWLRDRKRSPSQTAHYILKSLEDIDM	S266*
		NLGIRRESREIYRRLSDYSSHLPSEDGSQKQ	S588*
Q80VM3	Tetratricopeptide repeat protein 29	DNIYQLPDAEEETRRSPENQ___________	S467*
		ATEDNIYQLPDAEEETRRSPENQ________	T464*
Q6P902	Thioredoxin domain-containing protein 2	SSEDIIQSKKEDRPKSSEDIIQPKKEDRPKS	S221*
		SEDIIQSKKEDRPKSSEDIIQPKKEDRPKSS	S222*
		SSEDIIQSKKEDRPKSSEDIIQSKKEDRPKS	S176*
		SEDIIQSKKEDRPKSSEDIIQSKKEDRPKSS	S177*
Q715T0	Thioredoxin domain-containing protein 3	PHVRFAPVMIKKKRDSLQEYMDRQHMSDYCD	S270*
B1AZI6	THO complex subunit 2	IETNCLEEKSKRDYFTQLVLACLYLVSDTVL	T108*
		KSKRDYFTQLVLACLYLVSDTVLKERLDPET	Y116*
P97930	Thymidylate kinase	MEEKNLNWKVVDASKSIEEVHKEIRAHSEDA	Y32888*
A2ASS6	Titin	DRSGIRWIKCNKRRITDLRLRVTGLTEDHEY	T25663*
		SLVFSNGVERKDAGFYVVCAKNRFGIDQKTV	Y32888*
O08792	Transcription factor COE2	GTHYKLQLLYSNGVRTEQDLYVRLIDSVTKQ	T121*
		LQLLYSNGVRTEQDLYVRLIDSVTKQPIAYE	Y126*
Q99LC8	Translation initiation factor eIF-2B subunit alpha	EAVAAKKRFSVYITESQPDLSGKKMAKALSH	S155*
Q99P25	Translin-associated factor X-interacting protein 1	RDQFYNELQEIQRTSTPRPDWTKCESIIAGG	T364*
P0C1V4	Transmembrane and coiled-coil domain-containing protein 2	_______MPTFPTTTSSWDNLLNALARSSIW	S9*
		______MPTFPTTTSSWDNLLNALARSSIWN	S10*
Q14C59	Transmembrane protease serine 11B-like protein	KDPWSNKENLRRRIESILRQMLENNPESLTT	S134*
Q8BHM9	Transmembrane protease serine 11F	YLASFQVTSIKYRENYGIRSSREFIERSHQI	Y77*
Q9D6G5	Transmembrane protein 138	LFVFQRLAAVLYCYFYKRTAVRLGDPRFYQD	Y134*
Q9D9S2	Transmembrane protein 225	RCAMPRSIVHVHSVTSKDGSLNRPHTQARRV	S211*
Q9DA04	Transmembrane protein 89	RWRAKVAKGQLPAVTSSSGKHWKEQPTVSDR	S114*
		WRAKVAKGQLPAVTSSSGKHWKEQPTVSDRT	S115*
		RAKVAKGQLPAVTSSSGKHWKEQPTVSDRTL	S116*
		HRWRAKVAKGQLPAVTSSSGKHWKEQPTVSD	T113*
Q9D2H5	Tripartite motif-containing protein 42	YPIHSEMMIARKVTFSTHSFGNQQIYQRSSS	S523*
Q6PFY8	Tripartite motif-containing protein 45	______________MSEIRKPLLGFVHKLQD	S2*
Q924W6	Tripartite motif-containing protein 66	SGPAAESLGTRDGAESSLGNALCKMESEDCT	S785*
		GPAAESLGTRDGAESSLGNALCKMESEDCTR	S786*
Q9CWH5	tRNA (guanine(10)-N2)-methyltransferase homolog	MVPYLHSDSTYKIKIHTFNKTLTQEEKVKRI	S113*
Q0P5Y3	Tubulin polymerization-promoting protein family member 2	GIEGREETTDNSGYVSGYKGAGTYDKKNQ__	S157*
P70399	Tumor suppressor p53-binding protein 1	PNRTSSALAVTVEAASLPEEEKEEEELEEEK	S133*
P43404	Tyrosine-protein kinase ZAP-70	IPEGTKFDTLWQLVEYLKLKADGLIYRLKEV	Y238*

		THLPADSMRIVLERCYNDLRLLSMPSKTLKA	Y768*
Q8CB27	Ubiquitin thioesterase OTU1	VRCKAKGGTHLLQGLSSRTRLRELQGQIAAI	S59*
P62984	Ubiquitin-60S ribosomal protein L40;Ubiquitin;60S ribosomal protein L40;Ubiquitin-40S ribosomal protein S27a;Ubiquitin;40S ribosomal protein S27a;Polyubiquitin-B;Ubiquitin;Polyubiquitin-C;Ubiquitin;Ubiquitin-related 1;Ubiquitin-related 2	QQRLIFAGKQLEDGRTLSDYNIQKESTLHLV	T55*
P31254	Ubiquitin-like modifier-activating enzyme 1 Y	PVPKFAPKSGIRIHVSEQELQSTSATTIDDS	S809*
Q80Y39	Uncharacterized protein C10orf62 homolog	KRKAATETTEDKPLESHRANDSWIKSHFSRL	S23*
		SSMTKEMQRESGKSPSMEDDTWAAVAACTKE	S112*
		MLQRSTTHRRKGHAESRNISPEELKALEEVE	S154*
		NFLTHHETGVAGANQSHTVYSQSRHSNQSHH	S190*
		HETGVAGANQSHTVYSQSRHSNQSHHSYPSH	S195*
		LESHRANDSWIKSHFSRLSEERLPTYRYVSN	S36*
		LTHHETGVAGANQSHTVYSQSRHSNQSHHSY	T192*
		SRHGEVNTTLHVDTLTTKHGERGAALHRDSF	T73*
		RHGEVNTTLHVDTLTTKHGERGAALHRDSFA	T74*
Q05AH6	Uncharacterized protein C11orf84 homolog	DLQVIRVRMEEPPAVSLLQDWSKHPQGTKGV	S337*
E9QMW4	Uncharacterized protein C16orf96 homolog	TPKERVSYHKASKDVSPEDTGTKDEAPEVKV	S534*
Q5ND19	Uncharacterized protein C17orf47 homolog	GYYSSSQRATAAVSLSPPPLPSQRRAEATST	S57*
		PSPKILISEAENTMRSPTREREVTRKVTISP	S328*
		SPHHSPHHSPHRKNQSIQTMSSHLAGVHRNV	S133*
		PHHSPHRKNQSIQTMSSHLAGVHRNVSPVRE	S138*
Q8C5U4	Uncharacterized protein C17orf74 homolog	PRRGWGSLCKPVRLASNVGLWGRQGGILASL	S217*
Q8CIL4	Uncharacterized protein C1orf131 homolog	RERAIMLGAKPPKNTYVNYKVLQKQIKEKKI	Y195*
		AIMLGAKPPKNTYVNYKVLQKQIKEKKIAVE	Y198*
Q9CPZ3	Uncharacterized protein C1orf185 homolog	GNTVSNTFCSEQKTDSLKELLVLKNTEVGKH	S208*
Q9DAD0	Uncharacterized protein C1orf194 homolog	DPWSRLSSTPTATSRSRDTFFDSKIPKDDLD	S57*
		_______MLQPQETFSNPALRDEDKHLGNLW	S9*
		AHLAQQQDPWSRLSSTPTATSRSRDTFFDSK	T50*
		LAQQQDPWSRLSSTPTATSRSRDTFFDSKIP	T52*
A2AGB2	Uncharacterized protein C1orf87 homolog	TGVLERERARRLIHNYNLIYSLSLSPRRINQ	Y507*
		ERERARRLIHNYNLIYSLSLSPRRINQALQR	Y511*
		GEEEPQTPKPKPELPSHFYVRPVSPMDKHIK	S69*
		KPKPELPSHFYVRPVSPMDKHIKILPSPPVP	S77*
		TTSMKELLEGEEEPQTPKPKPELPSHFYVRP	T60*
Q8R3C1	Uncharacterized protein C2orf42 homolog	PLEITRSFIQNRDGTYELFKCPKVEVENIAE	Y468*
Q9DA60	Uncharacterized protein C2orf57 homolog	ADRLQDEGPRLRTVASLLRSARSAFSSGVMS	S468*
Q9DAG5	Uncharacterized protein C2orf61 homolog	LCPGQYEVLPAPVPKSPARSFVFRSSVQRFP	S153*
Q8CDN1		RKKDIGAKPKPVDDVSPQPVRARTPPENTVV	S258*

	Uncharacterized protein C3orf20 homolog	PKPVDDVSPQPVRARTPPENTVVELPDPCPE	T266*
		SQEAADGKGRLPDIASPSRDSPLNIKHKVAH	S111*
Q9DAF8	Uncharacterized protein C4orf51 homolog	DGKGRLPDIASPSRDSPLNIKHKVAHQIWGS	S116*
Q8CE72	Uncharacterized protein C5orf42 homolog	MIRWSDRRLLCDPGVTPSSCKYSPVIRVKTS	T1767*
Q3V037	Uncharacterized protein C6orf163 homolog	EFVTTGVTVIKDKKTSLGQLIKAKEHEMTIY	S205*
		RAAFQKYINYTFPRLSPGHADFILPERKKTP	S301*
Q9D5Y0	Uncharacterized protein C7orf31 homolog	DDLYWRQLEMKPLPISCSKSNHYIDYEPLKS	S453*
		LYWRQLEMKPLPISCSKSNHYIDYEPLKSAY	S455*
Q0VFX2	Uncharacterized protein C9orf117 homolog	QGKKIGGKKDASGTKTPELAMVEELKEFYHK	T30*
Q9CQC3	Uncharacterized protein C9orf135 homolog	__________MGTFDSQCHECFESKERWYEI	S6*
		__MGTFDSQCHECFESKERWYEIGPTDLLER	S14*
Q7TPM5	Uncharacterized protein C9orf9 homolog	SIEQVQSYMEHYCNNSTHRRILIMFMDICSE	S60*
Q3TTI8	Uncharacterized protein ENSP00000372125 homolog	RTREPFLKMIHAKESSPIYQTWTQREFLLPK	S35*
Q9D6A1	Unconventional myosin-Ih	ESGAGKTEASKKILQYFAVTCPMTESLQIAR	Y121*
Q9DAS2	UPF0573 protein C2orf70 homolog	LPPLSEKSKWNLLRLSPDYKRTYQTFPSGKR	S168*
Q9D131	UPF0686 protein C11orf1 homolog	ERYDLKNIVKPKPLPSQFGHAFETTYDANYS	S90*
Q6PE87	UPF0704 protein C6orf165 homolog	VYPLKEASTQSKREGSSRVPRPQIFIAGLRG	S587*
Q80Y73	UPF0722 protein C11orf88 homolog	_METGPRGCPSGRKESQEICSPGLLVFTGCS	S15*
		PYKPKDKVPKSKEVLSESGLRDQEEVKALE_	S154*
P62814	V-type proton ATPase subunit B, brain isoform	PDGTKRSGQVLEVSGSKAVVQVFEGTSGIDA	S92*
Q9D593	V-type proton ATPase subunit E 2	VMIVRCRPQDLHLVESAVLRAIPQYMRLCQK	S146*
Q8BMC1	V-type proton ATPase subunit G 3	______________MTSQSQGIQQLLQAEKR	T2*
Q91XD6	Vacuolar protein-sorting-associated protein 36	RLLLAEKMGHLCRDDSVEGLRFYPNLFMTQN	S371*
P70460	Vasodilator-stimulated phosphoprotein	RRPWEKNSTTLPRMKSSSSVTTSEAHPSTPC	S317*
		PWEKNSTTLPRMKSSSSVTTSEAHPSTPCSS	S319*
		WEKNSTTLPRMKSSSSVTTSEAHPSTPCSSD	S320*
Q9R1Z8	Vinexin	TWTKDSKRQDKRWVKYEGIGPVDESGMPIAP	Y170*
P63143	Voltage-gated potassium channel subunit beta-1	__MQVSIACTEHNLKSRNGEDRLLSKQSSNA	S14*
Q9D994	WD repeat-containing protein 38	ASGGWDKRAIVWEVQSGRRVHLLVGHCDSIQ	S141*
		VARSKCLHVLKGHQRSVETVSFSPDSKQLAS	S112*
		GRLLWRLAGHRGPVKSCCFSPDGRLIASSSS	S73*
		FSPDGRLIASSSSDHSIRLWDVARSKCLHVL	S91*
E9PYY5	WD repeat-containing protein 78	FTDVRVLRSTAEAAISKEELEKTIEIILTET	S188*
Q3U3V8	X-ray radiation resistance-associated protein 1	KVSFDTGLKKISRRDSQADVLGHILDRSFLL	S76*
Q9JJN2	Zinc finger homeobox protein 4	HLHSVSPDCVEKLLMTVPVPDVMMLNSMLLP	T1253*
Q8BI67	Zinc finger protein 473 homolog	RFSQNVYLQWHQKIHTGEKLCKTQSDSNLEG	T288*
Q6A085	Zinc finger protein 629	GKSFIRSSHLIQHRRTHTGEKPYKCPECGKS	T478*
		SFIRSSHLIQHRRTHTGEKPYKCPECGKSFS	T480*

Supplementary Data 3. Label-free quantitation of phosphopeptide level changes from capacitation time zero after BSA-induced capacitation by plotting the normalized ion intensities of tyrosine-phosphopeptides corresponding to identified phosphoproteins versus capacitation times (X-axis)

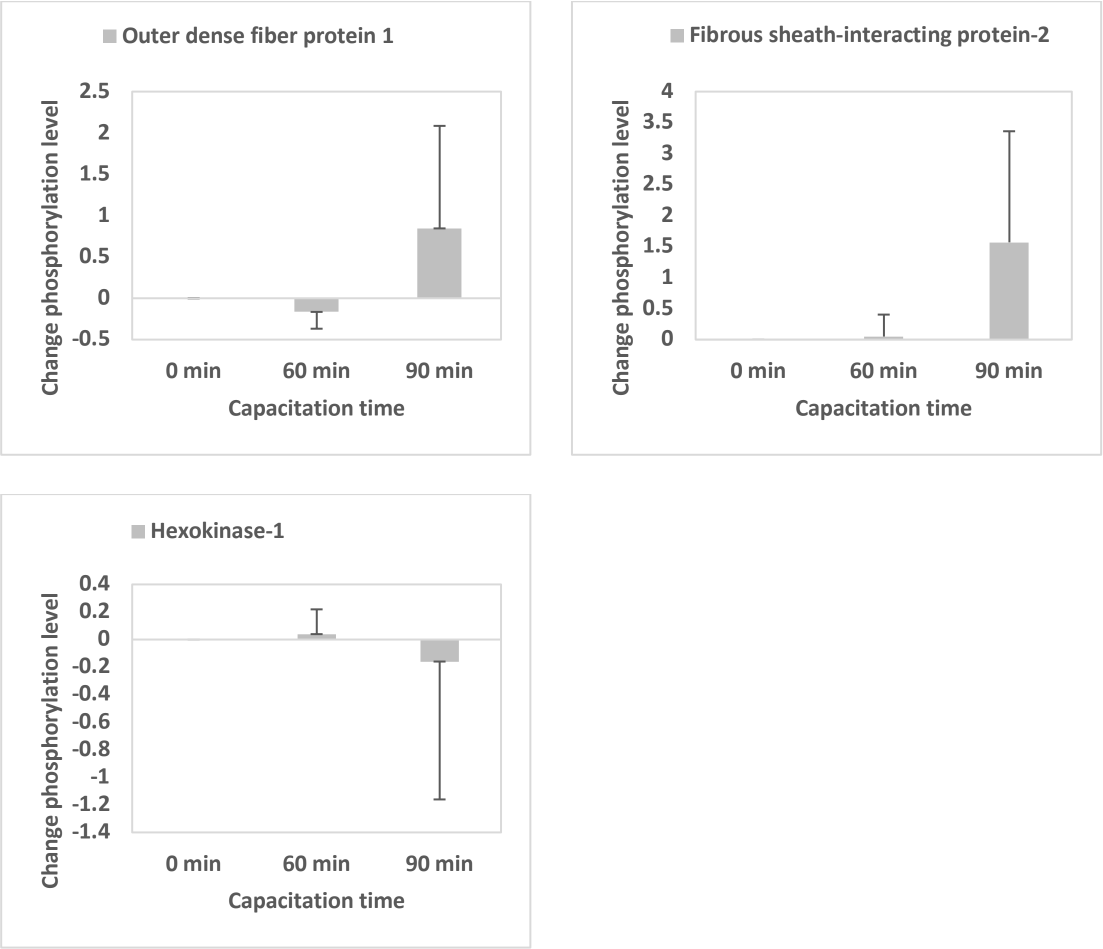

Supplementary Data 4. Different expressions of GSK-3 phosphorylation involved in GP6 signaling pathway generated through IPA canonical pathway analysis for comparison among all datasets (Cap 60/0, Cap 90/0, and Cap 90/60). (a) GSK-3 phosphorylation changes in the Cap 60/0 comparison (b) GSK-3 phosphorylation changes in the Cap 90/0 comparison (c) GSK-3 phosphorylation changes in the Cap 90/60 comparison. In the figure, red represents upregulation or increased expression; green indicated downregulation or decreased expression. The color intensity is proportional to the degree of fold change in protein expression. Direct and indirect interactions are indicated by solid, and dashed lines, respectively.

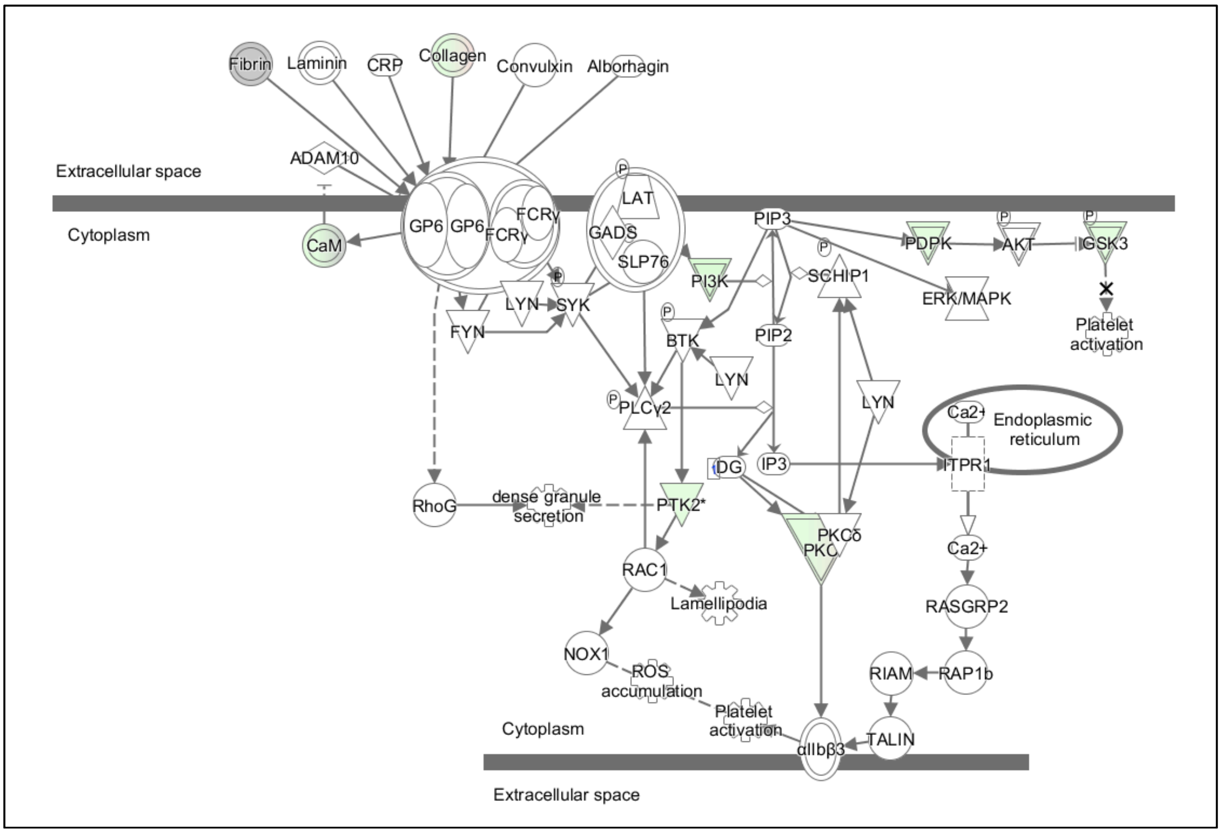

(a)

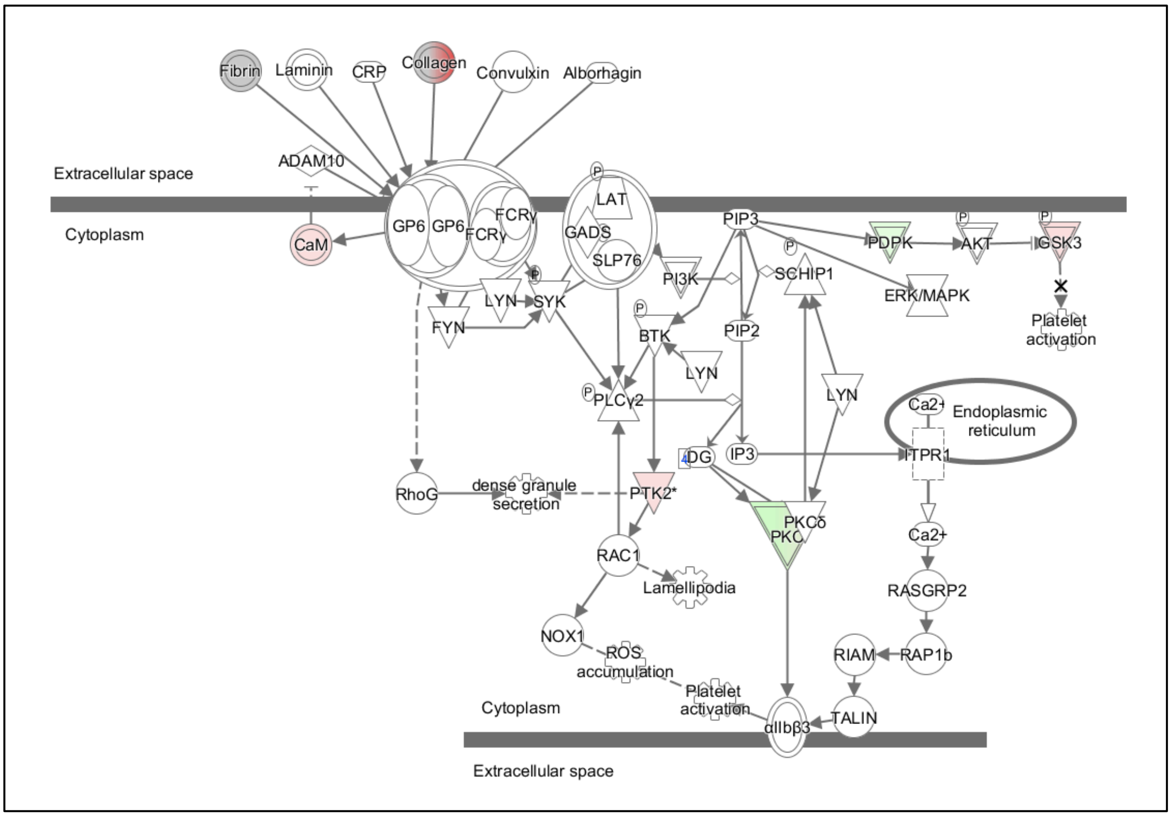

Fibrin
Laminin
CRP
Collagen
Convulxin
Alborhagin
ADAM10
Extracellular space
Cytoplasm
CaM
GP6
GP6
FCRγ
FCRγ
LAT
GADS
SLP76
PIP3
PDPK
AKT
GSK3
PI3K
SCHIP1
ERK/MAPK
LYN
SYK
FYN
BTK
PIP2
Platelet activation
LYN
PLCγ2
LYN
Ca2+
Endoplasmic reticulum
DG
IP3
ITPR1
RhoG
dense granule secretion
PTK2*
PKCδ
PKC
Ca2+
RAC1
Lamellipodia
RASGRP2
NOX1
ROS accumulation
RIAM
RAP1b
Platelet activation
Cytoplasm
αIIbβ3
TALIN
Extracellular space

(b)

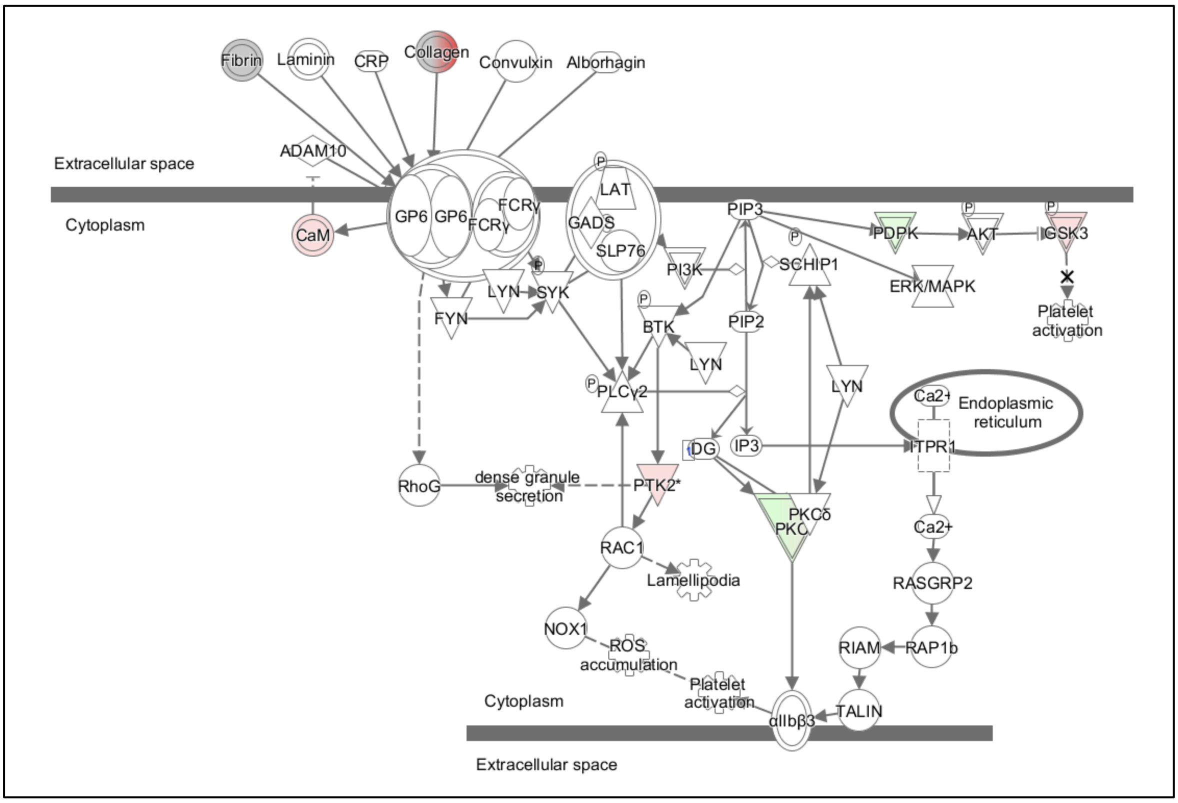

Fibrin
Laminin
CRP
Collagen
Convulxin
Alborhagin
ADAM10
Extracellular space
Cytoplasm
CaM
GP6
GP6
FCRγ
FCRγ
LAT
GADS
SLP76
PIP3
PDPK
AKT
GSK3
PI3K
SCHIP1
ERK/MAPK
LYN
SYK
FYN
BTK
PIP2
Platelet activation
LYN
PLCγ2
LYN
Ca2+
Endoplasmic reticulum
DG
IP3
ITPR1
RhoG
dense granule secretion
PTK2*
PKCδ
PKC
Ca2+
RAC1
Lamellipodia
RASGRP2
NOX1
ROS accumulation
RIAM
RAP1b
Platelet activation
Cytoplasm
αIIbβ3
TALIN
Extracellular space

(c)
